# Polystyrene–Poly(acrylic acid) Block Copolymers for Encapsulation of Butyrylcholinesterase into Injectable Nanoreactors

**DOI:** 10.3390/biom14121555

**Published:** 2024-12-05

**Authors:** Petr A. Fetin, Ivan M. Zorin, Zukhra M. Shaihutdinova, Patrick Masson, Tatiana N. Pashirova

**Affiliations:** 1 Department of Macromolecular Chemistry, Institute of Chemistry, St. Petersburg State University, 7/9 Universitetskaya nab, 199034 St. Petersburg, Russia; p.fetin@spbu.ru (P.A.F.); i.zorin@spbu.ru (I.M.Z.); 2 Institute of Fundamental Medicine and Biology, Kazan Federal University, 18 Kremlyovskaya St., 420008 Kazan, Russia; shajhutdinova.z@mail.ru; 3 Arbuzov Institute of Organic and Physical Chemistry, FRC Kazan Scientific Center of RAS, Arbuzov Str. 8, 420088 Kazan, Russia

**Keywords:** butyrylcholinesterase, bioscavenger, polymersomes, self-assembly, enzyme activity

## Abstract

The article is devoted to the creation of enzymatic nanoreactors based on polystyrene–block–poly(acrylic acid) (PS-b-PAA) copolymers containing bioscavengers capable of neutralizing toxic esters both in the body and in the environment. Block copolymers of different amphiphilicity, hydrophilicity and molecular weights were synthesized and characterized using gel permeation chromatography, NMR and UV spectroscopy. Polymeric nanocontainers in the absence and presence of human butyrylcholinesterase were made by film hydration and characterized by dynamic light scattering and microscopy methods. Enzyme activity was determined using the Ellman method. For the first time, factors that need to be taken into account for the creation of effective enzymatic nanoreactors based on PS-b-PAA are presented. The data obtained open up the possibility of PS-b-PAA nanoreactor use for future in vivo bioscavenger studies.

## 1. Introduction

Block copolymers with pronounced hydrophilicity and hydrophobicity of elementary blocks have a practical significance due to their ability to form vesicular-type molecular associates in solutions, called polymersomes or “microscopic sacs”(sac = bag) [1]. The vesicular morphology is achieved by maintaining the hydrophilic weight fraction between 25 and 45 wt % of the total polymer to ensure proper shape curvature of these associates [2,3,4,5]. They are quite stable and are widely used for the encapsulation of various compounds, including proteins and peptides, most often for medicinal uses [6,7,8] or immunotherapy [9]. The effect of using “a cargo” is usually achieved by its release or exchanging as a result of dilution, concentration or external stimuli responses. Another direction is the possibility of carrying out reactions of active substances inside containers with reactants coming from outside [10]. These active substances act as protein-containing functional modules [11,12], coined artificial cells [13,14,15]. To achieve this goal, polymer vesicles must be stable and must not release encapsulated active substances. This means that enzymes can be contained in ultra-small compartments or containers at concentrations down to the single molecule level [15]. At these scales, the local environment maintained by container boundaries and volume constraints can play an important role in reaction kinetics and mechanisms [16]. For example, enzyme catalytic efficiency (*k_cat_*/*K_m_*) can be significantly increased in confined space, in particular if there are high local concentrations of enzymes and substrates near the vesicle wall [17,18,19] or a crowded environment [20,21].

This work is aimed at the synthesis of relatively simple AB-type copolymers, consisting of two molecular blocks A and B, one (A) being hydrophilic and other (B) hydrophobic, and the production of two-phase polymer nanocompartments for the encapsulation of human butyrylcholinesterase (hBChE). Our group’s work focuses on the encapsulation of enzymes acting as organophosphate bioscavengers to produce nanoreactors for toxicant degradation [22,23]. Commonly used polymeric materials for vesicular containers include polystyrene, polybutadiene or hydrophobic poly(meth)acrylates as the membrane-forming unit and hydrophilic polymers such as polyethylene glycol or acrylic acid as corona-forming segments. In this work, we selected molecules capable of easily forming vesicles: polystyrene–block–poly(acrylic acid) (PS-b-PAA) copolymers. The self-organization of PS-b-PAA block copolymers has been intensively studied [24,25]. Usually, PS-b-PAA is dissolved in a good solvent for both blocks, such as water-miscible organic solvents (tetrahydrofuran, dimethyl sulfoxide, dimethylformamide or dioxane) before being transferred into an aqueous buffer to induce the formation of vesicular systems. To form reproducible morphologies of PS-b-PAA self-assemblies, careful control of parameters was required: solvent composition and water content, polymer structure and concentration and temperature [26,27,28]. Several kinds of cargo molecules were encapsulated into such PS-b-PAA vesicles [29,30]. It is important to mention that PS-b-PAA polymersomes are known to be very stable under normal conditions [25,31]; even at elevated temperatures, reduced pressure and different pH, and they do not release their cargo [29]. Stability is a very important characteristic of nanoreactors containing enzyme scavengers for circulating in the bloodstream for a long time and trapping toxicant molecules. To create stable and highly active enzymatic nanoreactors based on PS-b-PAA polymers, it is necessary to use mild methods such as film hydration, spraying and microfluidics, excluding the use of organic solvents that cause enzyme denaturation.

The PS-b-PAA block copolymers were obtained by the RAFT polymerization mechanism using two approaches: starting the synthesis from a polystyrene block or from a polyacrylate block. This makes it possible to obtain a representative series of block copolymers with different lengths of both blocks, as well as with different end groups. This provides the possibility of PS-b-PAA self-organization in water in the absence of any organic solvents and the encapsulation of hBChE, using the film hydration method to maintain the high catalytic activity of hBChE. 

## 2. Materials and Methods

### 2.1. Materials

RAFT agent 2-(dodecylthiocarbonothioylthio)-2-methylpropionic acid, DDMAT (Sigma-Aldrich, Saint-Louis, MO, USA), DMF (HPLC grade), ethanol, methanol and thionyl chloride were used without further purification. Azobisisobutyric acid dinitrile (AIBN) was recrystallized from ethanol. Styrene and acrylic acid were distilled in vacuo. Dioxane was distilled over granulated KOH. Benzene was distilled over sodium. Butyrylthiocholine iodide (BTC) and 5,5-dithiobis(2-nitrobenzoic acid) (DTNB) were from Sigma-Aldrich, Saint-Louis, MO, USA. Stock solutions of BTC (0.1 M) prepared in water were stored at −20 °C. All other chemicals and solvents were of chemical or biochemical grade. Ultra-purified water (18.2 MΩ cm resistivity at 25 °C) was produced from Direct-Q 5 UV equipment (Millipore S.A.S. 67120 Molsheim, France).

### 2.2. Synthesis of PS-b-PAA and PAA-b-PS Block Copolymers

**Figure 1:**
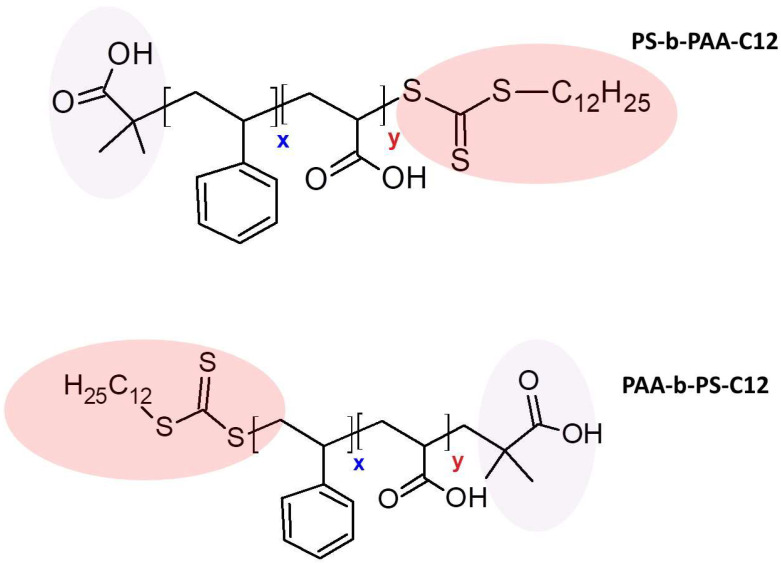
The structure of copolymers of series 1 (PS-b-PAA-C12) and series 2 (PAA-b-PS-C12).

The block copolymers were synthesized in two steps. First, macro-RAFT agents based on polystyrene or acrylic acid were synthesized by RAFT-polymerization. In the second step, the PS-b-PAA or PAA-b-PS block copolymers were prepared from the macro-RAFT agent. Even with equal degrees of polymerization of the styrene block and acrylic acid, these block copolymers differ in the order of terminal groups (Figure 1). The nature of the functional terminal group of the block has a significant effect on the properties of the copolymers in the studied range of molecular weights.

#### 2.2.1. Synthesis of Macro-RAFT Agent

Two macro-RAFT agents based on acrylic acid with different degrees of polymerization (*n* = 28, *n* = 57, see Table 1) and one based on styrene (*n* = 67) were obtained. The conditions to synthesize macro-RAFT agents were as follows.

- AA-RAFT agents

Acrylic acid 4 mL (4.86 M), AIBN (0.005 M), DDMAT (0.05 M) and dioxane 8 mL were mixed in an ampoule for polymerization. Before polymerization, the reaction mixture was degassed using a vacuum setup in three cycles: freezing in liquid nitrogen, vacuuming (2 mm Hg), thawing and then filling with argon. Synthesis took place in a sealed ampoule for 8 h at 80 °C. The polymer was isolated by lyophilization, dissolved in dioxane (10 mL) and then precipitated to acetone (200 mL). The polymer precipitated as a viscous gum, was dissolved in dioxane and then lyophilized. The theoretical degree of polymerization of acrylic acid under these conditions is *n* = 97, and *n* = 57, determined by ^1^H NMR. To obtain a low-molecular-weight macro-RAFT agent based on acrylic acid, a similar technique was used, but higher concentrations of DDMAT (0.1 M) and AIBN (0.01 M) were used. It was not possible to reprecipitate such a polymer from dioxane solution into acetone. Thus, low-molecular-weight impurities were extracted with an acetone/diethyl ether mixture. The theoretical degree of polymerization of acrylic acid under these conditions is *n* = 49, determined by the ^1^H NMR method to be *n* = 28.

- PS-RAFT agents

Styrene 8 mL (8.7 M), AIBN (0.005 M) and DDMAT (0.05 M) were mixed in an ampoule for polymerization. Before polymerization, the reaction mixture was degassed using a vacuum setup in three cycles: freezing in liquid nitrogen, vacuuming (2 mm Hg), thawing and then filling with argon. Synthesis took place in a sealed ampoule for 16 h at 80 °C. To remove unreacted styrene, we used triple dissolution of the product in benzene and subsequent freeze-drying of the product. The theoretical degree of polymerization of styrene under these conditions is *n* = 174 determined by GPC and *n* = 67 from ^1^H NMR, which in this case gave reduced degrees of polymerization due to a large error in integrating the end group of the polymer (Figure S1b).

#### 2.2.2. Synthesis Block Copolymers

- PS-b-PAA-C12 (Series 1)

A solution of PS-RAFT agent (1 g), acrylic acid (1 g) and AIBN (C = 0.001 M) in dioxane was prepared, the total volume was 4 mL, and then degassed. Synthesis was carried out in a sealed ampoule at a temperature of 80 °C in different periods of time: 3 h, 6 h and 24 h for 1-PS-b-AA-C12, 2-PS-b-AA-C12 and 3-PS-b-AA-C12, respectively. Depending on the molecular weight, the polymer was purified either by threefold lyophilization from dioxane or precipitation into diethyl ether followed by lyophilization from dioxane. They were dried to constant weight using an oil pump.

- PAA-b-PS-C12 (Series 2)

Precipitation of the PAA-RAFT agents was encountered when styrene was added to the reaction mixture; this drawback was overcome only by large dilution of the reaction participants. Three block copolymers of PAA-b-PS-C12 were obtained. Amounts of 2 mL styrene (for 1-PAA-b-PS-C12) and 4 mL styrene (for 2-PAA-b-PS-C12) were mixed with PAA-RAFT (*n* = 57, 2 g), AIBN (C = 0.001 M) and 8 mL dioxane. Before polymerization, the reaction mixture was degassed using a vacuum setup in three cycles: freezing in liquid nitrogen, vacuuming (2 mm Hg), thawing and then filling with argon. Synthesis took place in a sealed ampoule for 12 h at 80 °C. Amounts of 1.4 mL styrene, PAA-RAFT (*n* = 28, 2 g) and 9 mL dioxane were used in the case of 3-PAA-b-PS-C12. The polymer was isolated by freeze-drying from dioxane, low-molecular-weight impurities were removed by extraction with a mixture of acetone and diethyl ether and then the purified polymer phase was diluted with excess dioxane and isolated by freeze-drying.

4-PAA-b-PS-C12 and 5-PAA-b-PS-C12 were obtained by dispersion RAFT polymerization according to the adapted procedure from paper [32]. An amount of 0.5 g of PAA-RAFT agent (*n* = 28 (4-PAA-b-PS-C12) and *n* = 57 (5-PAA-b-PS-C12)) was dissolved in 11.2 mL methanol solution of AIBN C = 0.001 M and 0.005 M, correspondingly. An amount of 12.8 mL styrene was added in each flask. Adding styrene to a solution of macro-RAFT agent does not cause phase separation. The reaction mixture was degassed, using a vacuum setup in three cycles: freezing in liquid nitrogen, vacuuming (2 mm Hg), thawing and then filling with argon. Synthesis took place in a sealed ampoule for 8 h at 80 °C. After polymerization, the polymer was released as a separate phase dispersion (4-PAA-b-PS-C12) or macroscopic sediment (PAA-b-PS-C12). The reaction mixture was concentrated and diluted with benzene (4-PAA-b-PS-C12) or dioxane (5-PAA-b-PS-C12), and the product was isolated by freeze-drying.

#### 2.2.3. Hydrophobic End Group Removal in Block Copolymer

A 10–50-fold molar excess of AIBN was added to the copolymer solution in benzene and the mixture was kept at 80 °C for 24 h. After removing the trithiocarbonate group, the solubility of the polymers changed significantly. Then, the reaction mixture was precipitated in petroleum ether. The precipitate polymer was washed with benzene. Then, it was dissolved in DMF and placed inside a dialysis bag (cutoff 2000) and dialyzed against a DMF/methanol solution. The polymer solution was evaporated and dried. The dry polymer was subsequently dissolved in dioxane and lyophilized. 

### 2.3. Characterization of Block Copolymers

The degree of polymerization of PS and the polydispersity of both PS and diblock copolymers were determined by gel permeation chromatography (GPC). The polymers were denoted PSx-b-PAAy, where x and y stand for the degree of polymerization of the PS and PAA blocks, respectively. To determine the composition of the block copolymer and estimate the molecular weight, the method of end group analysis, using ^1^H NMR and UV spectroscopy were used (Table 1).

#### 2.3.1. Gel Permeation Chromatography (GPC)

GPC measurements were performed using a chromatograph (Shimadzu LC-20AD) equipped with a refractometric detector and TSKgel Guard, G5000HHR and G2500HHR columns (Tosoh Bioscience). Analysis conditions were as follows: 0.1 M LiBr in DMF, 60 °C and 0.75 mL/min. The polymer solution in the eluent (3 mg/mL) was filtered through membrane filters (PTFE, 0.45 μm, 50 mm). The average molecular weight (Mw) and dispersion index (Đ = Mw/Mn) were calculated according to the calibration curve in the Shimadzu LC solution v.1.25 software, using polystyrene standards. Before chromatographic analysis, in order to suppress the aggregation of PS-b-PAA copolymers under GPC conditions, the polyacrylic acid block was converted into methyl ester by the action of thionyl chloride in methanol according to the following procedure: SOCl_2_ was added dropwise to methanol (5 mL) at −18 °C, the copolymer (50 mg) was added to the methanol (1 mL), the mixture was kept for 1 hour upon cooling, then 1 hour at room temperature, and then 1 hour at methanol solution boiling temperature. The liquid phase was evaporated on a rotary evaporator, the polymer forming a transparent film on the flask walls. Then, the sample was dissolved in dioxane and isolated by lyophilization. As a result, a white free-flowing polymer powder was obtained. For GPC analysis, the sample (6 mg) was dissolved in 2 mL of eluent (0.1 M LiBr in DMF); the analysis was carried out 24 h after solution preparation. Samples were filtered through a syringe filter (PTFE, 0.22 μm) prior to analysis.

#### 2.3.2. ^1^H NMR Spectroscopy

^1^H NMR spectra were recorded on a Bruker Avance-400 spectrometer at 400 MHz. Chemical shifts were reported in ppm relative to residual signals of protons of deuterated solvents. CDCl_3_/ DMSO-*d_6_* were used as NMR solvents.

#### 2.3.3. UV–vis Spectroscopy 

UV–vis spectra were recorded in dioxane on a Shimadzu UV1600 spectrometer in 1 cm quartz cuvettes. 

### 2.4. Preparation of Empty and hBChE-Loaded PS-b-PAA Nanoreactors 

The thin-film hydration method was used for the preparation of solutions of PS-b-PAA copolymers. PS-b-PAA (0.02–0.5% *w*/*w*) was dissolved in 1 mL ethanol/chloroform (1:1) solution. The homogeneous polymer solution was kept for 3 h at 34 °C for preparing the thin film and then overnight for organic solvent evaporation. Pre-heated (37 °C) Tris buffer (10 mM, pH 7.4) or water was added to rehydrate the thin film of block copolymers in the absence or presence of hBChE. The solution was stirred under magnetic stirring (600 rpm) (Ika, Germany) for 3 h at 37 °C, and then for 5 h at room temperature.

### 2.5. Characterization of Empty and hBChE-Loaded PS-b-PAA Nanoreactors

#### 2.5.1. Dynamic Light Scattering 

Mean particle size, zeta potential and the polydispersity index were determined using a Malvern Instrument Zetasizer Nano (Worcestershire, UK) and Brookhaven 90Plus Nanoparticle Size Analyzer (Holtsville, NY, USA). All samples were analyzed in triplicate. 

#### 2.5.2. Transmission Electron Microscopy

TEM images were obtained using a microscope Hitachi HT7700, Tokyo, Japan. The images were acquired at an accelerating voltage of 110 kV. Samples was added to 300-mesh copper grids with continuous carbon–formvar support films and dried at room temperature for 3 h.

#### 2.5.3. Atomic Force Microscopy

Samples (15 μL) were applied on a freshly cleaved mica (5 × 5 mm plate), aged for 60 s and then spin-coated at 3000 rpm for 60 s. They were stored in air for no less than 24 h. AFM images were captured with a Veeco diNanoForce V (Veeco, Santa-Barbara, CA, USA) operating in tapping mode (TM). The AFM probe NT-MDT HA_NC Etalon (NT-MDT, Zelenograd, Russia), a resonant frequency of 224 kHz and a force constant of 10 N were used, with a scan rate of 1 Hz. Images were processed with Veeco Nanoscope Analysis 1.2 software.

### 2.6. Butyrylcholinesterase Preparation

An HBChE tetramer (340 kDa) from pooled Cohn fraction IV-4 from plasma from blood donors was a gift from Prof. O. Lockridge (University of Nebraska Medical Center, Omaha, NE, USA). The enzyme was highly purified to homogeneity by affinity chromatography on Hupresin gel [33]. The specific activity of the preparation with 1 mM BTC was 3386 units/mL phosphate buffer at 25 °C (one unit corresponds to the number of micromoles of BTC hydrolyzed per minute, 6.8 mg/mL, i.e., 0.5 units/μg).

### 2.7. BChE Activity Measurements

Standard BChE activity was measured by the Ellman method [34] with 1 mM BTC as the substrate in 0.1 M phosphate buffer pH 7.0 at 25 °C. A thermostated double-beam spectrophotometer at λ = 412 nm was used.

For steady-state kinetic analysis of free and encapsulated BChE, kinetics were measured over a wide range of BTC concentrations (5 to 10,000 μM). Catalytic parameters were determined using the Radic equation (Equation (1)), which conveniently describes the catalytic behavior of BChE with positively charged substrates like BTC [35].

(1) $$v=\left(\frac{k_{\mathrm{cat}}\left[E\right]}{1+K_{m}/\left[S\right]}\right)\left(\frac{1+{b\left[S\right]/K}_{ss}}{1+{\left[S\right]/K}_{ss}}\right)$$

where *k_cat_* is the catalytic constant (= Vmax/[*E*]), [*E*] is the active site concentration, *K_m_* is the Michaelis constant, *K_ss_* is the dissociation constant of the second substrate molecule bound to the peripheral anionic site (PAS) and b is a factor (>1) expressing the enzyme activation upon binding of the second substrate molecules to the PAS. Experiments were performed in triplicate and rate data (Equation (1)) fitted with OriginPro 8.5 (Originlab Co., Northampton, MA, USA). 

### 2.8. Determination of Inhibition Constants and Type of Reversible Inhibition

Kinetic studies of BChE reversible inhibition by polymeric nanoparticles or water–organic solution (7.5 %vol. methanol or 2% vol. DMSO) of polymers were performed with three concentrations of BTC (0.1, 0.5 and 1 mM) as the substrate in 0.1 M phosphate buffer, pH 7.0 at 25 °C and λ = 412 nm. Assays were initiated by the addition of the enzyme. The final concentration of the enzyme was 0.05 nM. Inhibition constants and type of reversible inhibition were determined according to the graphical methods of Cornish-Bowden [36] by building Dixon plots (1/V vs. polymer concentration) and Cornish-Bowden plots (S/V vs. polymer concentration). Experiments were performed in triplicate and rate data fitted with OriginPro 8.5 (Originlab Co., Northampton, MA, USA). 

### 2.9. Time-Dependent Progressive Inhibition of BChE

Time-dependent progressive inhibition of BChE was performed by mixing BChE (0.625 µM) with solutions of polymeric nanoparticles or water–organic solutions of polymers (1 mg/mL) and incubating the mixture up to 24 h. During incubation, aliquots were taken at different times and residual enzyme activity was immediately measured with 1 mM BTC as the substrate in 0.1 M phosphate buffer pH 7.0 at 25 °C. The final concentration of BChE in the cuvette was 0.05 nM. Residual activity was calculated as the ratio of activity at time point τ to activity at time zero (Vi/Vo × 100%). Rate data were fitted with OriginPro 8.5 (Originlab Co., Northampton, MA, USA).

### 2.10. Recovery of Activity

The dilution method was used to estimate the reversibility or irreversibility of BChE inhibition. The solution of 3-PAA-b-PS polymeric nanoparticles was diluted in the range of 5 to 500 times in 0.1 M phosphate buffer pH 7.0 at 25 °C. Activity was measured with 1 mM of BTC right after dilution to estimate the recovery of activity after dilution. There was control of the dilution factor by measuring the activity of the uninhibited enzyme with the same dilutions. Rate data were fitted with OriginPro 8.5 (Originlab Co., Northampton, MA, USA).

### 2.11. Self-Assembly of PS-b-PAA

#### 2.11.1. Tensiometry

Surface tension measurements were performed using the du Nouy ring detachment method (Kruss K6 Tensiometer, Hamburg, Germany). Temperature was kept constant at 25 °C during all measurements.

#### 2.11.2. Conductometry

Specific conductivity was measured using a WTW InoLabCond 720 precision conductivity meter (WTW Gmb, Weilheim, Germany). Reproducibility was checked for samples, and no significant differences were observed. All samples were studied at constant temperature, 25 °C. 

#### 2.11.3. UV Spectrophotometry (Dye Solubilization)

Solubilization of the dye (Sudan I) was performed by adding an excess of crystalline Sudan I to the solutions of polymers. These solutions were allowed to equilibrate for about 48 h at constant temperature (25 °C), followed by filtration. UV absorbance was measured using a PerkinElmer λ35 (PerkinElmer Instruments, Shelton, CT, USA) at 485 nm (for Sudan I). Quartz cuvettes (L = 0.2 cm) containing samples were used.

## 3. Results

### 3.1. Synthesis of Polystyrene–Block–Poly(Acrylic Acid) Copolymers (PS-b-PAA)

Block copolymers of the AB type were obtained by RAFT polymerization. There are two possible ways to synthesize such block copolymers by the RAFT mechanism: (i) starting from the polystyrene block; (ii) starting from the polyacrylic acid block. When using asymmetric RAFT agents, the block copolymers synthesized starting from polystyrene or polyacrylic acid will differ in the terminal groups belonging to the hydrophobic and hydrophilic blocks, respectively (Figure 1). We first synthesized a styrene-based macro-RAFT agent corresponding to the hydrophobic block A of the block copolymer. Then, this macro-RAFT agent was introduced into a reaction with acrylic acid. Polymerization was carried out for different times and block B of different lengths was built up; thus, copolymers of series 1 (PS-b-PAA) were obtained. 

In contrast to the synthesis of series 1 copolymers, where dioxane was the solvent of the reaction, it was difficult to select a common solvent for the synthesis of series 2 copolymers (PAA-b-PS). Styrene is a precipitant for polyacrylic acid; adding styrene to a solution of the macro-RAFT agent in dioxane resulted in phase separation. In this regard, a large volume of solvent had to be added to homogenize the reaction mixture. This resulted in the yield of such polymerization not exceeding 10%. To increase the length of the styrene block, controlled suspension polymerization described in [32] was used. Methanol was used as a solvent for the synthesis; as the length of the polystyrene block increased, the copolymer began to form a separate phase, but did not lose the ability to attach a monomer. This approach made it possible to obtain block copolymers with a high degree of styrene polymerization and, accordingly, high yields.

The next step was aimed at synthesizing block copolymers without a terminal long-chain dodecyl group. The dodecyl hydrophobic group can contribute to the polymers’ ability to bind to the enzyme, thus affecting the enzyme activity. For this purpose, two methods were used: the polymers were treated with either alkali or a large excess of free radicals generated by the thermal decomposition of AIBN. The latter method turned out to be more effective. The scheme of block copolymer synthesis is presented in Figure 1. 

The first approach is based on hydrolysis in the presence of nucleophilic agents. The implementation of this route seems obvious and was the simplest. However, the acrylic acid block complicates the hydrolysis due to salt formation and polymer precipitation in the mixed solvents dioxane/water and DMF/water. Therefore, the use of NaOH and KOH was impossible, since the heterogeneous hydrolysis mode in the case of polymers obviously leads to incomplete conversion of functional groups and the formation of a difficult-to-separate mixture of macromolecules. Polymer precipitation was also observed in the case of introducing polymers in dioxane, THF or DMF into a reaction with an alcoholic solution of trimethylamine, as well as diisopropylethylamine. Only in the dioxane/triethylamine system was precipitation not observed and hydrolysis successfully implemented. 

The second approach, free radical cleavage–recombination under the action of a large excess of free radicals, was successful. The products of trithiocarbonate group transformation were removed from the polymer, as confirmed by UV spectroscopy (Figure S1). The difference in solubility of the block copolymers before and after the transformation of the end group also indirectly indicates the occurrence of a reaction. 

Then, all obtained block copolymers were analyzed by several physicochemical methods. All characteristics are presented in Table 1. 

**Figure 2:**
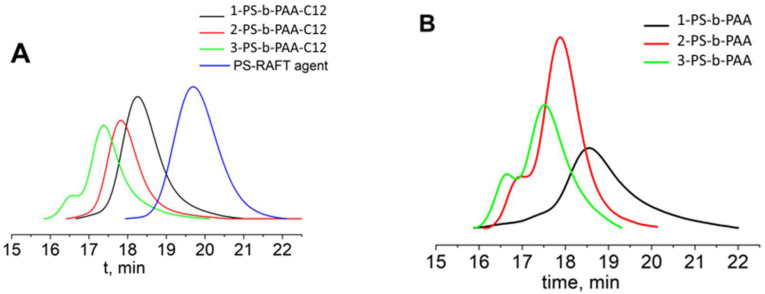
GPC chromatograms of (**A**) PS-b-PAA-C12 and (**B**) PS-b-PAA. TSKgel Guard, G5000HHR and G2500HHR columns (Tosoh Bioscience). Analysis conditions: 0.1 M LiBr in DMF, 60 °C and 0.75 mL/min.

Molecular weight determination of amphiphilic block copolymers, although widespread, is in fact a non-trivial task precisely because of the amphiphilic nature of the investigated objects. Therefore, two spectral methods of analysis by end groups (UV–vis and ^1^H NMR) as well as GPC were used (Figure 2). The degree of polymerization of the polyacrylic acid block was determined (i) from NMR data, using the ratio of signals of the chain, the phenyl group and the methyl group of the RAFT agent (Figure S2); (ii) using UV spectroscopy data, namely the absorption of the trithiocarbonate group at 310 nm; (iii) from GPC data, where the carboxyl groups of polyacrylic acid were preliminarily converted into methyl ester to avoid possible polymer aggregation. The peak shapes in GPC chromatograms were not asymmetric (Figure 2B). This may indicate partial cross-linking of macromolecules. However, the correspondence of the obtained molecular weights with the characteristics of the original polymers (before transformation of the trithiocarbonate group (Table 1)), as well as the low dispersion indices for these polymers, indicates that the fraction of cross-links is minor and has little effect on the physicochemical properties of the products.

### 3.2. Encapsulation of hBChE into Nanoreactors Based on Polystyrene–Block–Poly(Acrylic Acid) Copolymers

All the obtained block copolymers are poorly soluble in water. Therefore, aqueous solutions of all the block copolymers were prepared by the film rehydration method, using our slightly modified technique [22,23]. The temperature (from 25 to 55 °C), concentration (between 0.05 and 0.5% *w*/*w*) and environment of solution preparation (water and buffer) were varied. Dynamic light scattering (Table 2) and high-resolution transmission electron microscopy were used to characterize the physicochemical properties of the nanoreactors.

**Figure 3:**
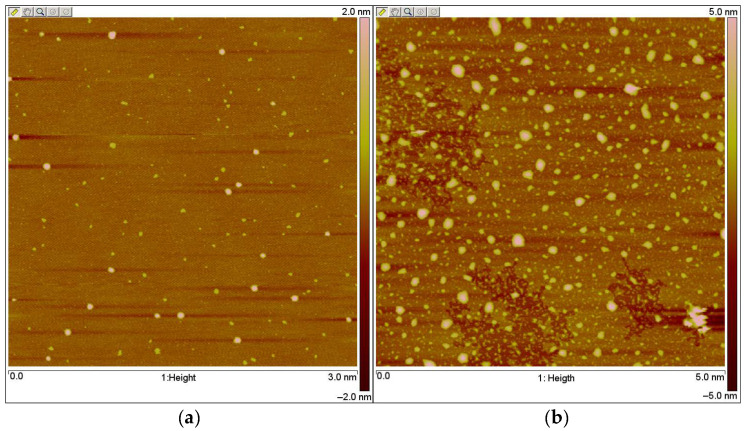
AFM imaging of 1-PS-PAA (**a**) and 3-PS-PAA (**b**), C_Polymers_ = 0.1%, water, 25 °C.

High polydispersity was observed for samples with a C12 long chain. The highest polydispersity ≤ 0.8 was for 1-PS-b-PAA-12. Particles prepared with block copolymers without a tail demonstrate lower polydispersity. Extrusion using polycarbonate membranes allows a reduction in polydispersity to 0.2 and below. The sizes are presented with a distribution by number, since the size with a distribution by intensity is basically the same. In our opinion, the largest size is mainly due to the agglomeration of nanoparticles. The particle sizes based on PS-b-PAA “without tail” are about 60 nm and they are smaller compared to PS-b-PAA-C12. Increasing the polyacrylic acid chain and molecular weight in series PS-b-PAA has almost no effect on the size of nanoparticles in the absence of the enzyme. Figure 3 and Figure S3 show that for the PS-b-PAA series, the size of nanoparticles without the enzyme ranges from about 16 to 117 nm. The particles present in the solution are mainly spherical. Spherical mono-macromolecular particles also present in the solution cannot significantly contribute to scattering in the presence of the largest particles. The particle size, according to qualitative estimates, is in the range of 50–100 nm. These values do not contradict the DLS data. Therefore, a quantitative assessment of the size of micelles or vesicles from AFM data could be misleading, and thus is not recommended.

On the contrary, for the series PAA-b-PS, the polymer size increases three times with an increasing chain length of polystyrene; the size of the nanoparticles decreases three times with an increasing molecular weight. The highest size nanoparticles of about 280 nm, 250 nm and 130 nm are observed for 3-PAA-b-PS, 4-PAA-b-PS and 5-PAA-b-PS, respectively. Large sizes of assemblies may indicate the formation of structures of layered- or vesicular-type. The calculation of the hydrophilic weight fraction between 25 and 45 wt % of the total polymer could help theoretically to recognize the vesicular morphology of nanoparticles. 

**Figure 4:**
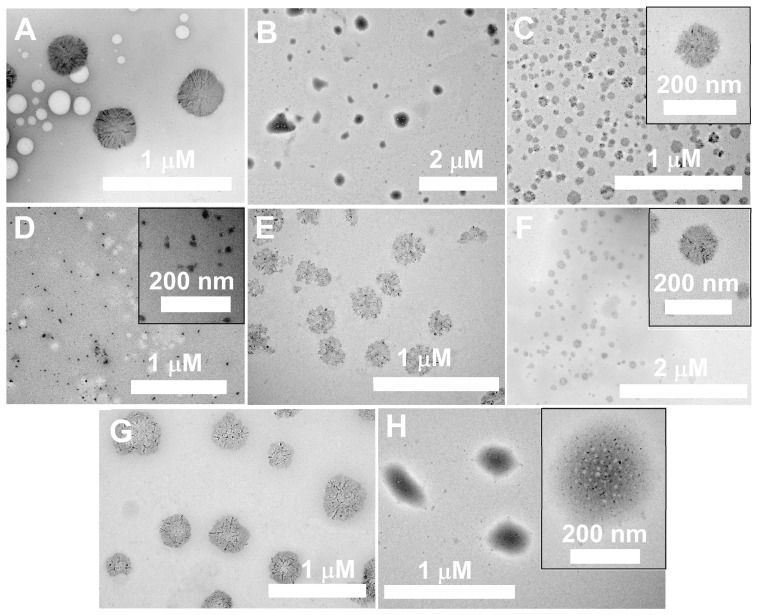
TEM imaging of BChE-loaded 1-PS-b-PAA (**A**), 2-PS-b-PAA (**B**), 3-PS-b-PAA (**C**), 1-PAA-b-PS (**D**), 2-PAA--b-PS (**E**), 5-PAA-b-PS (**F**), 3-PAA-b-PS (**G**) and 4-PAA-b-PS (**H**), C_Polymers_ = 0.01 μg/mL, 10 mM TrisHCl buffer, 25 °C. Scale bar is 1 or 2 μm and 200 nm is in insert (**C**,**D**,**F**,**H**).

Interestingly, this self-organizing behavior of block copolymers is changed by the encapsulation of an enzyme into the polymeric nanoparticles. Encapsulation of BChE (57 μg/mL) into the polymeric nanoparticles promotes an increase in the sizes for all polymeric bodies except for 1-PAA-b-PS. The greatest increase in size from 60 nm to 180 nm is observed for the BChE-1-PS-b-PAA enzyme–polymer associations. The size of assemblies BChE-3-PAA-b-PS and BChE-5-PAA-b-PS increases by 1.5 times. DLS data for enzyme-loaded nanoparticles are confirmed by TEM (Figure 4 and Figures S4–S11). For the “tailless” PS-b-PAA series, an increase in the PAA chain length and molecular weight of the polymers leads to a decrease in size of enzyme–polymer assemblies (moving from A to C in Figure 4). For the PAA-b-PS series, an increase in PS chain length (moving from D to F in Figure 4) leads to an increase in size of the enzyme–polymer assemblies. An increase in the molecular weight of PAA-b-PS polymers (from Figure 4G to H and F) leads to a decrease in size. The morphology of all nanoparticles is close to spherical shape. 

### 3.3. Kinetic Studies of BChE with Nanoreactors Based on Polystyrene–Block–Poly(Acrylic Acid) Copolymers

The study of BChE activity was carried out in three cases: (1) the enzyme was encapsulated in polymeric particles; (2) the enzyme in was in water-buffered solution of polymeric nanoparticles prepared by the thin-film rehydration method; and (3) the enzyme was in water-buffered organic solutions of block copolymers.

#### 3.3.1. BChE Loaded into Polymeric Nanoparticles

**Figure 5:**
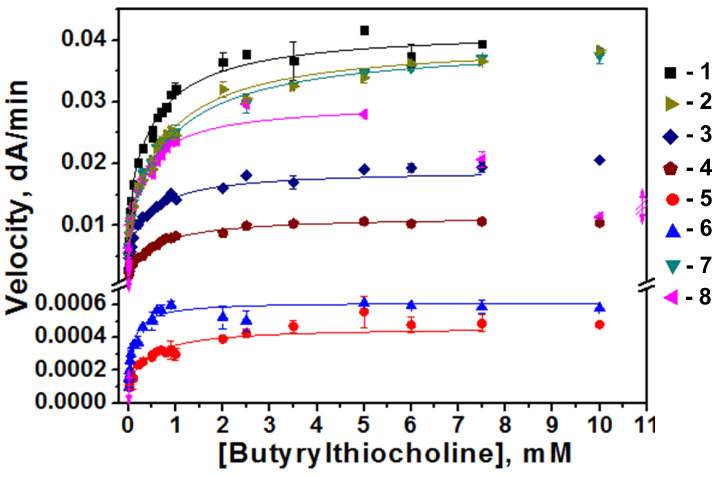
Dependence of initial rates of butyrylthiocholine hydrolysis by free BChE (1) loaded into polymeric nanoparticles 1-PS-b-PAA (2), 2-PS-b-PAA (3), 3-PS-b-PAA (4), 1-PAA-b-PS (5), 2-PAA-b-PS (6), 4-PAA-b-PS (7) and 5-PAA-b-PS (8) on BTC concentration in phosphate buffer 0.1 M, pH 7.0, C_BChE_ = 0.05 nM, C_polymer_ = 8 µg/mL in cuvette, 25 °C.

The enzyme activity was monitored at pH 7.0 with BTC as the substrate. Encapsulation of the enzyme into 2-PS-b-PAA-C12 and 3-PS-b-PAA-C12 nanoparticles results in a progressive decrease in enzyme activity by 50% and 76%, respectively, but over time (24 h or more) no enzyme activity change was observed. A similar loss of activity after 24 h was observed after the encapsulation of the enzyme by 3-PAA-b-PS. Therefore, the activity of the enzyme encapsulated into nanoparticles was subsequently studied after 24 h. Dependences of rates of BTC hydrolysis (Velocity, dA/min) by the enzyme are presented in Figure 5.

To evaluate the effect of encapsulation on enzyme activity, the catalytic parameters of free BChE and BChE loaded into polymeric nanoparticles were determined by Equation (1) (Table 3). 

All polymers interact with BChE and alter the catalytic parameters, except *k_cat_*/*K_m_*. Interestingly, *k_cat_*/*K_m_* values do not change for nanoparticles that have structures close to polymersomes. The fact that *k_cat_*/*K_m_* does not change indicates a parallel decrease in both *k_cat_* and *K_m_*_,_ as for uncompetitive inhibition. Dixon plots and Cornish-Bowden plots clarify this point [36]. As a consequence, at a very low substrate concentration ((S)<<K_m_), the catalytic behavior of BChE in the presence of polymers is the same as the free enzyme. 

The catalytic constant (*k_cat_*) and dissociation constants (*K_m_* and *K_ss_*) are decreased for the PS-b-PAA polymer series with an increase in the acrylic acid polymer chain and molecular weight at the same time, indicating an increase in affinity for both the PAS and catalytic active site (CAS). For the PAA-b-PS polymer series, the catalytic constant (*k_cat_*) increases with an increase in the chain length of polystyrene and the hydrophilic/hydrophobic balance. Changes in other catalytic parameters for this series of polymers do not depend on polymer structure. 

The b factor is not altered, indicating that the molecular machinery between the PAS and CAS is not altered (the omega loop that connects the PAS and CAS is functional) [35]. 

In the next experiments, only some types of polymers were used when the encapsulation process significantly affected the catalytic activity (*k_cat_*/*K_m_*). It is important to understand what the cause is, either (i) this block copolymer acts as an inhibitor or (ii) mixed polymer–enzyme assemblies form.

#### 3.3.2. BChE in Water–Organic and Polymeric Nanoparticle Solutions

In the next second step, the solution of prepared polymer nanoparticles (without enzyme) was added to the enzyme solution. Then, the kinetics were studied at the time point τ = 0 and also during their joint incubation over time. BChE activity significantly decreased after 15 min of incubation in the 2-PS-b-PAA-C12 and 3-PS-b-PAA-C12 nanoparticle solutions. BChE activity comprised 95, 82 and 10% after incubation in 2-PS-b-PAA-C12 solution at 0.24, 0.4 and 4 mg/mL polymer, respectively, and 97 and 12% in 3-PS-b-PAA-C12 solution at 0.24 and 4 mg/mL, respectively. The structure of these polymers contains a long chain. Therefore, a strong decrease in activity can be caused by the formation of micellar assemblies of these polymers. In the next experiment, these polymers were used to study their self-assembly behavior.

Dixon plot and Cornish-Bowden plot analyses (Figures S12–S19) of BChE reversible inhibition by polymers show that weak inhibition is observed for all block copolymers (Table 4). However, inhibition is non-competitive, indicating that weak polymer binding does not affect substrate binding. At these concentrations, the interaction should not affect the catalytic properties of the enzyme.

**Figure 6:**
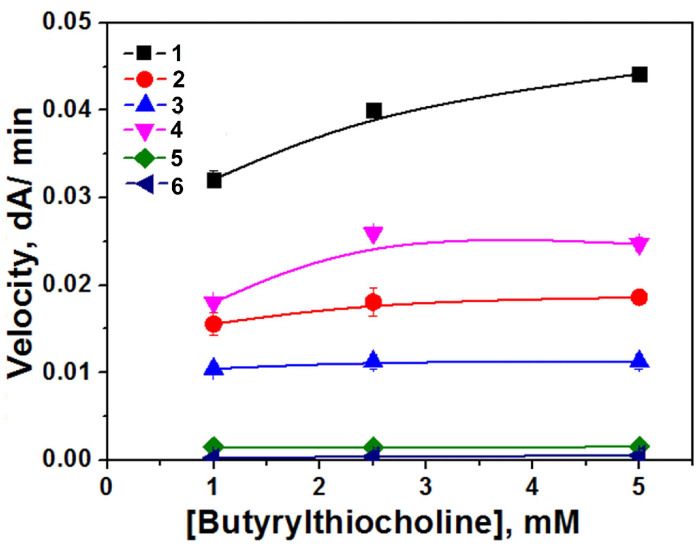
Dependence of initial rates of butyrylthiocholine (BTC) hydrolysis by free BChE (1) and BChE loaded into polymeric nanoparticles of 1-PAA-b-PS at different concentrations (% *w*/*w*): 0.02 (2), 0.04 (3), 0.05 (4), 0.075 (5) and 0.1 (6) on BTC concentration in 0.1 M phosphate buffer, pH 7.0, C_BChE_ = 0.4 nM, 25 °C.

For further explanation regarding the decrease in enzyme catalytic activity after encapsulation by 1-PAA-b-PS, which is not an inhibitor, the enzyme was loaded into nanoparticle solutions with different polymer concentrations (Figure 6). As we see, the enzyme lost activity in polymeric nanoparticle solutions for all concentrations. 

**Figure 7:**
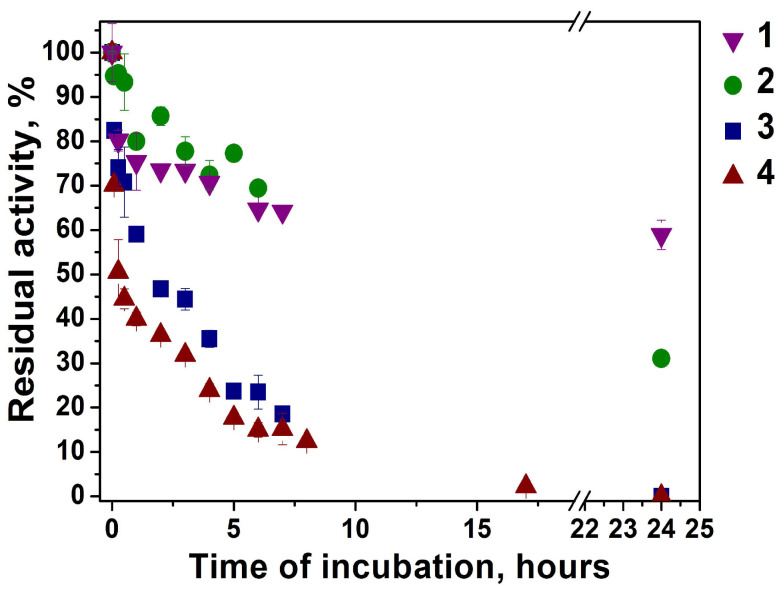
Butyrylthiocholine hydrolysis by BChE in polymeric nanoparticle solution of 1-PAA-b-PS (1), water–organic (2) polymeric nanoparticle solution of 3-PAA-b-PS (3) and polymeric nanoparticle solution of 3-PAA-b-PS with addition of free BChE (4) in phosphate buffer 0.1 M, pH 7.0, C_BChE_ = 0.05 nM, 25 °C.

Figure 7 shows the results of enzyme activity incubated in block copolymer solutions over time. The incubation of enzyme in 1-PAA-b-PS solution shows a weak decrease in enzyme activity. The maintenance of enzyme activity over time and the effect of increasing the polymer concentration on enzyme activity indicate the influence of polymer aggregates on enzyme catalysis. As can be seen in Table 2, the aggregate size for this polymer is the smallest and the size decreases in the presence of BChE (Figure 3b). This may support the statement of interaction between polymer aggregates and the enzyme. 

The 3-PAA-b-PS nanoparticles show a progressive decay in BChE activity with full loss of activity of the enzyme after 24 h of incubation. It was of importance to determine whether this inhibition was reversible or irreversible. The dilution method showed that there was no spontaneous recovery of activity after diluting sample, indicating irreversible inhibition (Figures S20 and S21). 

### 3.4. Self-Assembly Study of PS-b-PAA-C12

Conductometry and tensiometry methods did not reveal polymer aggregation in solution (Figures S22 and S23). Detection of micelle formation was possible using a more traditional method for investigating micellar polymer aggregates, namely solubilization of the hydrophobic probe dye Sudan I. The absorption spectra of Sudan I in block copolymer water solutions with an increase in their concentration are shown in Figure S24. Absorption at λ = 485 nm increases with increasing 2-PS-b-PAA-C12 and 3-PS-b-PAA-C12 concentration (Figure S25). This confirms the solubilization of the hydrophobic dye at low concentrations (0.05 mg/mL). This also indicates the formation of hydrophobic domains in block copolymer solution already at this concentration. A sharp increase in Sudan I solubilization above 1 g/L is observed for both 2-PS-b-PAA-C12 and 3-PS-b-PAA-C12. This indicates the formation of micellar-type aggregates capable of solubilizing the hydrophobic probe in the micellar (hydrophobic) core. It should be noted that the slope of absorption dependence on 2-PS-b-PAA-C12 concentration is greater than for 3-PS-b-PAA-C12, indicating greater solubilization capacity compared to 3-PS-b-PAA-C12. This is likely due to the more favorable hydrophilic–lipophilic balance of 2-PS-b-PAA-C12 for the formation of a more capacious solubilizing core (assemblies of 2-PS-b-PAA-C12 are larger than those of 3-PS-b-PAA-C12).

## 4. Discussion

Usually, self-assembly structures of synthetic block copolymer amphiphiles are determined by their hydrophilic weight fraction. As a rule, increasing this parameter favors structures with a higher curvature, such as rod-like and spherical micelles. Consequently, an increase in PAA chain length leads to a decrease in the size of enzyme–polymer assemblies (moving from A to C in Figure 4). An increase in PS chain length in the PAA-b-PS series (moving from D to F in Figure 4) leads to an increase in size and the formation of aggregates with a lower curvature, such as vesicles. 

Theoretically, the hydrophilic weight fraction for 1-PS-b-PAA, 1-PAA-b-PS and 2-PAA-b-PS is between 25 and 45 weight % so that they can organize the vesicular morphology. However, the self-assembly morphology can be tuned by minute (micro and millimolar) amounts of additives (acids, bases or inorganic salts), especially in the cases of ionic hydrophilic blocks [25,37]. The strength of repulsive interactions among PAA chains decreases and the stretching of the PS chains increases. Then, the aggregates change their morphology from spherical to rodlike, and from rodlike to bilayer [38]. Interestingly, for polymers 1-PS-b-PAA (Figure 4A), 4-PAA-b-PS (Figure 4H) and 5-PAA-b-PS (Figure 4F), the particle structures are very close to the vesicles that we already observed for polymersomes of PEG-PS in previous works [22,23,39]. It may be stated that the morphology of nanoparticles made of 1-PAA-b-PS, 2-PAA-b-PS, 4-PAA-b-PS and 5-PAA-b-PS was changed due to multiple interactions between BChE and PAA chains, and a decrease in the strength of repulsive interactions among PAA chains.

The overall opposite inhibitory effects of PAA-PS vs. PS-PAA correlate with the respective hydrophilic/hydrophobic balance of these polymers (in the PS-PAA series, there is increasing hydrophilicity, while in the PAA-PS series there is increasing hydrophobicity). Moreover, the size of enzyme–nanoreactors made with these polymers shows the same opposite trend (Figure 4). It is noteworthy that the highest *k_cat_* of the enzyme is for the largest nanoreactor sizes (Figure 4, panel A vs. panel F). Also, the opposite inhibitory effects of 1-PS-PAA and 2-PAA-PS of similar Xn PS and XnPAA (Table 1) on *k_cat_* and *K_m_* (Table 3) are similar to the opposite catalytic behavior of enzymes, like BChE and trypsin, with normal-type substrates and “reverse” substrates [40,41]. Because of the strict stereospecificity of ligand/substrate binding, any change in spatial relationships between the enzyme active surface and interacting molecules will alter the enzyme behavior. In particular, the reverse structure of ligands/substrates dramatically affects both binding and catalytic processes. 

Thus, polymers interact only with the enzyme surface, bind on a site distinct from the PAS, but close, and, thus, alter the affinity for the substrate at low and high concentrations (increase in affinity). This increase in substrate affinity indicates a conformational change that improves the tight adjustment between substrate and binding sites (PAS and CAS). The fact that *k_cat_* is decreased suggests that the binding of polymers on the surface alters the overall molecular dynamics of the enzyme. The image of polymers anchored at several points on the enzyme surface and acting as a net that restrains enzyme molecular motion may be exact. Such a situation was observed with the inhibition of acetylcholinesterase by a polymeric toxin from a marine sponge [42]. 

It is clearly seen that the activity of enzyme decreases in polymeric solution over time. This behavior is likely due to the formation of mixed polymer–enzyme assemblies (Figure 4G) rather than sealed enzyme nanoreactors. This may take place due to the low molecular weight of 3-PAA-PS compared to other polymers (3-PAA-PS, c.f. Table 1). The high hydrophilicity of BChE (25% of glycans on surface [43] imposes a curvature on the organized structure that prevents the formation of a vesicular structure of nanoreactors). TEM data (Figure 4G) confirm the formation of mixed enzyme–polymer complexes. In such structures, the enzyme activity was impaired. This effect does not depend on reduced diffusion of the substrate toward the enzyme active site, but it is rather due to reversible inhibition of the enzyme and blockade of the peripheral active site entrance by polymeric chains. Indeed, the polymer in solution is an uncompetitive inhibitor and empty nanoparticles made with this polymer act as mixed-type inhibitors of BChE (Table 4). 

Slow irreversible inhibition of BChE (Figure 7) may be due to polymeric nanoparticle-induced partial enzyme unfolding or the result of polymer anchoring on multiple binding sites, which impairs the enzyme molecular dynamics and, therefore, the catalytic activity after the initial fast reversible inhibition step. Such slow-binding inhibition (called slow-binding inhibition of type B) of BChE is rare but has been described, in particular with bulky ligands [44].

## 5. Conclusions

This study demonstrates that the use of block copolymers based on polyacrylic acid and polystyrene for encapsulating enzymes and making nanoreactors with high activity implies that it is necessary to carefully select the polymer structure and to take into account several factors: (1) the hydrophilicity parameter of the polymer to obtain vesicular structures to prevent enzyme inhibition/inactivation; (2) the absence of lipophilic long-chain fragments in the structure of polymers to minimize the formation of micellar aggregates; (3) polymers of low molecular weight capable of forming mixed-block polymer–enzyme assemblies must not be used; and (4) the formation of block copolymer self-assemblies with a size smaller than 30 nm is not suitable for preparation of nanoreactors. These constraints are of importance for the medical use of BChE-based nanoreactors for the inactivation of toxic compounds, where the enzyme acts either as a stoichiometric or a catalytic bioscavenger [16,35]. Therefore, the present study indicates that polymers 1-PS-b-PAA, 4-PAA-b-PS and 5-PAA-b-PS are suitable polymers for making enzyme nanoreactors of a polymersome structure to be used for this purpose.

## Figures and Tables

**Table 1:** Characteristics of block copolymers with series 1 (PS-b-PAA) and 2 (PAA-b-PS).

Types of Copolymers	Xn PS	Xn PAA	Mn	Mw/Mn
PS-RAFT	67 *	-	7300	1.05
Series 1				
1-PS-b-PAA-C12	67 *	66	13,000	1.08
2-PS-b-PAA-C12	67 *	102	16,100	1.08
3-PS-b-PAA-C12	67 *	162	21,200	1.16
1-PS-b-PAA	67 *	43	11,000	1.22
2-PS-b-PAA	67 *	125	18,300	1.15
3-PS-b-PAA	67 *	180	23,000	1.19
Series 2				
PAA-RAFT	-	28 **		
PAA-RAFT	-	57 **		
1-PAA-b-PS	55 *	57 **	18,800	1.22
2-PAA-b-PS	68 *	57 **	20,100	1.22
3-PAA-b-PS	3 *	28 **	6500	1.16
4-PAA-b-PS	144 *	28 **	21,200	1.2
5-PAA-b-PS	223 *	57 **	36,200	1.27

* GPC; ** ^1^H NMR.

**Table 2:** Empty and BChE-loaded nanoreactor characteristics, where size is hydrodynamic diameter (particle number distribution), PDI is polydispersity index and ξ or zeta potential is electrokinetic potential. The medium was phosphate and TrisHCl buffer, 10 mM, pH = 7.4, 25 °C.

Nanoreactors	C_polym_ (%, *w*/*w*)	Size (nm)	PDI	ξ (mV)
2-PS-b-PAA-C12	0.05	91 ± 22	0.56 ± 0.03	-
	0.5	91 ± 25	0.41 ± 0.02	-
BChE-2-PS-b-PAA-C12	0.5	91 ± 9	0.40 ± 0.02	-
3-PS-b-PAA-C12	0.5	68 ± 16	0.6 ± 0.003	-
BChE-3-PS-b-PAA-C12	0.5	79 ± 18	0.67 ± 0.02	
1-PS-b-PAA	0.1	62 ± 2	0.3± 0.01	−31.0 ± 1
BChE-1-PS-b-PAA	0.1	183 ± 70	0.35± 0.05	−25.4 ± 1
2-PS-b-PAA	0.1	51 ± 10	0.36 ± 0.01	−29.0 ± 1
BChE-2-PS-b-PAA	0.1	65 ± 30	0.35 ± 0.02	−16.4 ± 1
3-PS-b-PAA	0.1	59 ± 9	0.34 ± 0.02	−29.0 ± 1
BChE-3-PS-b-PAA	0.1	98 ± 50	0.34 ± 0.04	−24.0 ± 1
1-PAA-b-PS	0.1	28 ± 5	0.44 ± 0.03	−24.0 ± 2
BChE-1-PAA-b-PS	0.1	24 ± 10	0.38 ± 0.02	-
2-PAA-b-PS	0.1	23 ± 6	0.39 ± 0.01	−19.2 ± 1
BChE-2-PAA-b-PS	0.1	32 ± 10	0.38 ± 0.01	-
3-PAA-b-PS	0.1	278 ± 30	0.32 ± 0.01	−22.0 ± 1
BChE-3-PAA-b-PS	0.1	438 ± 20	0.27 ± 0.01	-
4-PAA-b-PS	0.1	243 ± 70	0.32 ± 0.02	−25.0 ± 1
BChE-4-PAA-b-PS	0.1	257 ± 70	0.32 ± 0.02	
5-PAA-b-PS	0.1	87 ± 20	0.30 ± 0.02	−35.5 ± 1
BChE-5-PAA-b-PS	0.1	125 ± 60	0.25 ± 0.1	

**Table 3:** Catalytic parameters (±SE) for BChE-catalyzed hydrolysis of BTC in phosphate buffer, 0.1 M, pH 7.0, 25 °C.

Nanoreactors	Vmax × 10^2^ (dA/min)	*k_cat_* _(_min^−1^)	*K_m_* _(_μM)	*k_cat_*/*K_m_* × 10^−9^ (M^−1^min^−1^)	*K_ss_* (μM)	*b*
BChE	1.57 ± 0.07	23,200 ± 1000	10.9 ± 1.1	2.1 ± 0.3	680 ± 50	2.7 ± 0.1
BChE-2-PS-b-PAA-C12	No activity	-	-	-	-	-
BChE-3-PS-b-PAA-C12	No activity	-	-	-	-	-
BChE-1-PS-b-PAA	1.08 ± 0.15	16,000 ± 2000	7.7 ± 2.8	2.1 ± 1.0	880 ± 130	3.7 ± 0.5
BChE-2-PS-b-PAA	0.68 ± 0.03	10,200 ± 400	8.2 ± 1.1	1.2 ± 0.2	530 ± 60	2.8 ± 0.1
BChE-3-PS-b-PAA	0.32 ± 0.03	4800 ± 400	2.1 ± 1.0	2.3 ± 1.3	700 ± 80	3.6 ± 0.3
BChE-1-PAA-b-PS	0.012 ± 0.001	180 ± 20	1.4 ± 1.2	0.13 ± 0.12	470 ± 70	3.8 ± 0.4
BChE-2-PAA-b-PS	0.029 ± 0.004	430 ± 50	10.3 ± 1.8	0.04 ± 0.01	180 ± 50	2.1 ± 0.3
BChE-3-PAA-b-PS	No activity	-	-	-	-	-
BChE-4-PAA-b-PS	1.09 ± 0.07	16,100 ± 1000	5.7 ± 1.1	2.8 ± 0.7	1000 ± 75	3.6 ± 0.2
BChE-5-PAA-b-PS	1.12 ± 0.07	16,500 ± 1000	6.9 ± 1.6	2.4 ± 0.7	480 ± 50	2.7 ± 0.2

**Table 4:** Inhibition of BChE by polymeric nanoparticles and polymers in water–organic solution.

Type of Copolymers	K_i_ (mg/mL)	K′_i_ (mg/mL)	Type of Inhibition
2-PS-b-PAA-C12 *	7.09 ± 1.04	10.02 ± 0.82	Mixed
3-PS-b-PAA-C12 *	12.65 ± 3.08	12.27 ± 3.36	Non-competitive
1-PS-b-PAA	-	-	Not inhibitor
2-PS-b-PAA	0.15 ± 0.04	-	Competitive
3-PS-b-PAA	-	1.65 ± 0.11	Uncompetitive
1-PAA-b-PS	-	-	Not inhibitor
3-PAA-b-PS	1.49 ±0.76	2.54 ±0.42	Mixed
3-PAA-b-PS *	-	2.12 ± 0.49	Uncompetitive

* water–organic solutions.

## Data Availability

The original contributions presented in this study are included in the article/Supplementary Materials. Further inquiries can be directed to the corresponding authors.

## Supplementary Materials

The following supporting information can be downloaded at: https://www.mdpi.com/article/10.3390/biom14121555/s1, Figure S1. UV Absorbance spectra of 1-PS-b-PAA and 3-PS-b-PAA in DMF; Figure S2. 1H NMR spectra of macro-RAFT agent based on acrylic acid and styrene; Figure S3. AFM imaging of 1-PS-b-PAA and 3-PS-b-PAA; Figure S4. TEM imaging of BChE-loaded 1-PS-b-PAA polymer; Figure S5. TEM imaging of BChE-loaded 2-PS-b-PAA polymer; Figure S6. TEM imaging of BChE-loaded 3-PS-b-PAA polymer; Figure S7. TEM imaging of BChE-loaded 1-PAA-b-PS polymer; Figure S8. TEM imaging of BChE-loaded 2-PAA-b-PS polymer; Figure S9. TEM imaging of BChE-loaded 3-PAA-PS polymer; Figure S10. TEM imaging of BChE-loaded 4-PAA-b-PS polymer; Figure S11. TEM imaging of BChE-loaded 5-PAA-b-PS polymer; Figure S12. Dixon plot and Cornish-Bowden plot analyses of inhibition of BChE by 2-PS-b-PAA-C12; Figure S13. Dixon plot and Cornish-Bowden plot analyses of inhibition of BChE by 3-PS-b-PAA-C12; Figure S14. Dixon plot and Cornish-Bowden plot analyses of inhibition of BChE by 1-PS-b-PAA; Figure S15. Dixon plot and Cornish-Bowden plot analyses of inhibition of BChE by 2-PS-b-PAA; Figure S16. Dixon plot and Cornish-Bowden plot analyses of inhibition of BChE by 3-PS-b-PAA; Figure S17. Dixon plot and Cornish-Bowden plot analyses of inhibition of BChE by 1-PAA-b-PS; Figure S18. Dixon plot and Cornish-Bowden plot analyses of inhibition of BChE by 3-PAA-b-PS; Figure S19. Dixon plot and Cornish-Bowden plot analyses of inhibition of BChE by 3-PAA-b-PS, Figure S20. Progressive curves of 3-PS-b-PAA after dilution; Figure S21. Progressive curves of free BChE after dilution; Figure S22. Surface tension of 2-PS-b-PAA-C12 and 3-PS-b-PAA-C12 solutions; Figure S23. Specific conductivity of 2-PS-b-PAA-C12 and 3-PS-b-PAA-C12 solutions; Figure S24. Absorption spectra of Sudan I in 2-PS-b-PAA-C12 and 3-PS-b-PAA-C12 solutions; Figure S25. Absorption of Sudan I at λ = 485 nm in 2-PS-b-PAA-C12 and 3-PS-b-PAA-C12 solutions. Table S1 Empty nanoreactor characteristics determined by DLS method at different temperature; Table S2. Empty nanoreactor characteristics determined by DLS method in water.

## References

[1] Discher D.E., Eisenberg A. (2002). Polymer vesicles. Science.

[2] Discher D.E., Ahmed F. (2006). Polymersomes. Annu. Rev. Biomed. Eng..

[3] Ahmed F., Discher D.E. (2004). Self-porating polymersomes of PEG–PLA and PEG–PCL: Hydrolysis-triggered controlled release vesicles. J. Control. Release.

[4] Valentini M., Napoli A., Tirelli N., Hubbell J.A. (2003). Precise Determination of the Hydrophobic/Hydrophilic Junction in Polymeric Vesicles. Langmuir.

[5] Velluto D., Demurtas D., Hubbell J.A. (2008). PEG- b -PPS Diblock Copolymer Aggregates for Hydrophobic Drug Solubilization and Release: Cyclosporin A as an Example. Mol. Pharm..

[6] Hua C., Qiu L. (2024). Polymersomes for Therapeutic Protein and Peptide Delivery: Towards Better Loading Properties. Int. J. Nanomed..

[7] Gouveia M.G., Wesseler J.P., Ramaekers J., Weder C., Scholten P.B.V., Bruns N. (2023). Polymersome-based protein drug delivery—Quo vadis?. Chem. Soc. Rev..

[8] Lo C.H., Zeng J. (2023). Application of polymersomes in membrane protein study and drug discovery: Progress, strategies, and perspectives. Bioeng. Transl. Med..

[9] Slezak A., Chang K., Hossainy S., Mansurov A., Rowan S.J., Hubbell J.A., Guler M.O. (2024). Therapeutic synthetic and natural materials for immunoengineering. Chem. Soc. Rev..

[10] Wang Y., Zhao Q., Haag R., Wu C. (2022). Biocatalytic Synthesis Using Self-Assembled Polymeric Nano- and Microreactors. Angew. Chem. Int. Ed..

[11] Hirschi S., Ward T.R., Meier W.P., Müller D.J., Fotiadis D. (2022). Synthetic Biology: Bottom-Up Assembly of Molecular Systems. Chem. Rev..

[12] Gaur D., Dubey N.C., Tripathi B.P. (2022). Biocatalytic self-assembled synthetic vesicles and coacervates: From single compartment to artificial cells. Adv. Colloid Interface Sci..

[13] Jiang W., Wu Z., Gao Z., Wan M., Zhou M., Mao C., Shen J. (2022). Artificial Cells: Past, Present and Future. ACS Nano.

[14] Kim H., Yeow J., Najer A., Kit-Anan W., Wang R., Rifaie-Graham O., Thanapongpibul C., Stevens M.M. (2022). Microliter Scale Synthesis of Luciferase-Encapsulated Polymersomes as Artificial Organelles for Optogenetic Modulation of Cardiomyocyte Beating. Adv. Sci..

[15] Zong W., Shao X., Li J., Chai Y., Hu X., Zhang X. (2023). Synthetic Intracellular Environments: From Basic Science to Applications. Anal. Chem..

[16] Shajhutdinova Z., Pashirova T., Masson P. (2022). Kinetic Processes in Enzymatic Nanoreactors for In Vivo Detoxification. Biomedicines.

[17] Ranquin A., Versées W., Meier W., Steyaert J., Van Gelder P. (2005). Therapeutic Nanoreactors: Combining Chemistry and Biology in a Novel Triblock Copolymer Drug Delivery System. Nano Lett..

[18] Chen Q., Schönherr H., Vancso G.J. (2009). Block-copolymer vesicles as nanoreactors for enzymatic reactions. Small.

[19] Chen Q., Rausch K.G., Schönherr H., Vancso G.J. (2010). α-Chymotrypsin-Catalyzed Reaction Confined in Block-Copolymer Vesicles. ChemPhysChem.

[20] Baumann P., Spulber M., Fischer O., Car A., Meier W. (2017). Investigation of Horseradish Peroxidase Kinetics in an “Organelle-Like” Environment. Small.

[21] Schvartzman C., Zhao H., Ibarboure E., Ibrahimova V., Garanger E., Lecommandoux S. (2023). Control of Enzyme Reactivity in Response to Osmotic Pressure Modulation Mimicking Dynamic Assembly of Intracellular Organelles. Adv. Mater..

[22] Pashirova T., Shaihutdinova Z., Mansurova M., Kazakova R., Shambazova D., Bogdanov A., Tatarinov D., Daudé D., Jacquet P., Chabrière E. (2022). Enzyme Nanoreactor for In Vivo Detoxification of Organophosphates. ACS Appl. Mater. Interfaces.

[23] Pashirova T., Shaihutdinova Z., Tatarinov D., Mansurova M., Kazakova R., Bogdanov A., Chabrière E., Jacquet P., Daudé D., Akhunzianov A.A. (2023). Tuning the Envelope Structure of Enzyme Nanoreactors for In Vivo Detoxification of Organophosphates. Int. J. Mol. Sci..

[24] Zhang L., Eisenberg A. (1995). Multiple Morphologies of “Crew-Cut” Aggregates of Polystyrene-b-poly(acrylic acid) Block Copolymers. Science.

[25] Shen H., Eisenberg A. (1999). Morphological Phase Diagram for a Ternary System of Block Copolymer PS 310—b-PAA 52 /Dioxane/H 2 O. J. Phys. Chem. B.

[26] Luo L., Eisenberg A. (2001). Thermodynamic size control of block copolymer vesicles in solution. Langmuir.

[27] Lim Soo P., Eisenberg A. (2004). Preparation of block copolymer vesicles in solution. J. Polym. Sci. Part B Polym. Phys..

[28] Luo L., Eisenberg A. (2002). One-Step Preparation of Block Copolymer Vesicles with Preferentially Segregated Acidic and Basic Corona Chains. Angew. Chem. Int. Ed..

[29] Haas S., Chen Y., Fuchs C., Handschuh S., Steuber M., Schönherr H. (2013). Amphiphilic Block Copolymer Vesicles for Active Wound Dressings: Synthesis of Model Systems and Studies of Encapsulation and Release. Macromol. Symp..

[30] Choucair A., Lim Soo P., Eisenberg A. (2005). Active Loading and Tunable Release of Doxorubicin from Block Copolymer Vesicles. Langmuir.

[31] Zhang L., Yu K., Eisenberg A. (1996). Ion-Induced Morphological Changes in “Crew-Cut” Aggregates of Amphiphilic Block Copolymers. Science.

[32] He W.-D., Sun X.-L., Wan W.-M., Pan C.-Y. (2011). Multiple Morphologies of PAA- b -PSt Assemblies throughout RAFT Dispersion Polymerization of Styrene with PAA Macro-CTA. Macromolecules.

[33] Schopfer L.M., David E., Hinrichs S.H., Lockridge O. (2023). Human butyrylcholinesterase in Cohn fraction IV-4 purified in a single chromatography step on Hupresin. PLoS ONE.

[34] Ellman G.L., Courtney K.D., Andres V., Featherstone R.M. (1961). A new and rapid colorimetric determination of acetylcholinesterase activity. Biochem. Pharmacol..

[35] Masson P., Shaihutdinova Z., Lockridge O. (2023). Drug and pro-drug substrates and pseudo-substrates of human butyrylcholinesterase. Biochem. Pharmacol..

[36] Cornish Bowden A. (1974). A simple graphical method for determining the inhibition constants of mixed, uncompetitive and non competitive inhibitors. Biochem. J..

[37] Liu F., Eisenberg A. (2003). Preparation and pH Triggered Inversion of Vesicles from Poly(acrylic Acid)-b lock-Polystyrene- b lock -Poly(4-vinyl Pyridine). J. Am. Chem. Soc..

[38] Zhang L., Eisenberg A. (1996). Morphogenic Effect of Added Ions on Crew-Cut Aggregates of Polystyrene-b-poly(acrylic acid) Block Copolymers in Solutions. Macromolecules.

[39] Pashirova T., Shaihutdinova Z., Tatarinov D., Titova A., Malanyeva A., Vasileva O., Gabdurakhmanov K., Dudnikov S., Schopfer L.M., Lockridge O. (2024). Pharmacokinetics and fate of free and encapsulated IRD800CW-labelled human BChE intravenously administered in mice. Int. J. Biol. Macromol..

[40] Nozawa M., Tanizawa K., Kanaoka Y. (1980). ‘Inverse’ substrates for butyrylcholinesterase. Biochim. Biophys. Acta—Enzymol..

[41] Tanizawa K., Kanaoka Y., Lawson W.B. (1987). Inverse substrates for trypsin and trypsin-like enzymes. Acc. Chem. Res..

[42] Sepčić K., Marcel V., Klaebe A., Turk T., Šuput D., Fournier D. (1998). Inhibition of acetylcholinesterase by an alkylpyridinium polymer from the marine sponge, Reniera sarai. Biochim. Biophys. Acta—Protein Struct. Mol. Enzymol..

[43] Boyko K.M., Baymukhametov T.N., Chesnokov Y.M., Hons M., Lushchekina S.V., Konarev P.V., Lipkin A.V., Vasiliev A.L., Masson P., Popov V.O. (2019). 3D structure of the natural tetrameric form of human butyrylcholinesterase as revealed by cryoEM, SAXS and MD. Biochimie.

[44] Masson P., Lushchekina S.V. (2016). Slow-binding inhibition of cholinesterases, pharmacological and toxicological relevance. Arch. Biochem. Biophys..

