# Supplementary material for: Polystyrene–Poly(acrylic acid) Block Copolymers for Encapsulation of Butyrylcholinesterase into Injectable Nanoreactors
Source: Biomolecules. 2024 Dec 5;14(12):1555. doi: 10.3390/biom14121555 (PMC11673596; doi:10.3390/biom14121555)
Supplement: Supplementary file 1 [file biomolecules-14-01555-s001.zip › biomolecules-3322745-supplementary.pdf]

Supplementary Materials

Contents

Figure S1. UV Absorbance spectra of 1-PS-b-PAA and 3-PS-b-PAA in DMF S3
Figure S2. ^{1}H NMR spectra of macro-RAFT agent based on acrylic acid and styrene S4
Table S1. Empty nanoreactor characteristics determined by DLS method at different temperature S5
Figure S3. AFM imaging of 1-PS-b-PAA and 3-PS-b-PAA S6
Figure S4. TEM imaging of BChE-loaded 1-PS-b-PAA polymer S7
Figure S5. TEM imaging of BChE-loaded 2-PS-b-PAA polymer S8
Figure S6. TEM imaging of BChE-loaded 3-PS-b-PAA polymer S9
Figure S7. TEM imaging of BChE-loaded 1-PAA-b-PS polymer S10
Figure S8. TEM imaging of BChE-loaded 2-PAA-b-PS polymer S11
Figure S9. TEM imaging of BChE-loaded 3-PAA-PS polymer S12
Figure S10. TEM imaging of BChE-loaded 4-PAA-b-PS polymer S13
Figure S11. TEM imaging of BChE-loaded 5-PAA-b-PS polymer S14
Figure S12. Dixon plot and Cornish-Bowden plot analyses of inhibition of BChE by 2-PS-b-PAA-C12 S15
Figure S13. Dixon plot and Cornish-Bowden plot analyses of inhibition of BChE by 3-PS-b-PAA-C12 S16
Figure S14. Dixon plot and Cornish-Bowden plot analyses of inhibition of BChE by 1-PS-b-PAA S17
Figure S15. Dixon plot and Cornish-Bowden plot analyses of inhibition of BChE by 2-PS-b-PAA S18
Figure S16. Dixon plot and Cornish-Bowden plot analyses of inhibition of BChE by 3-PS-b-PAA S19
Figure S17. Dixon plot and Cornish-Bowden plot analyses of inhibition of BChE by 1- PAA-b-PS S20
Figure S18. Dixon plot and Cornish-Bowden plot analyses of inhibition of BChE by 3-PAA-b-PS S21
Figure S19. Dixon plot and Cornish-Bowden plot analyses of inhibition of BChE by 3-PAA-b-PS S22
Figure S20. Progressive curves of 3-PS-b-PAA after dilution S23
Figure S21. Progressive curves of free BChE after dilution S24
Table S2. Empty nanoreactor characteristics determined by DLS method in water S25
Figure S22. Surface tension of 2-PS-b-PAA-C12 and 3-PS-b-PAA-C12 solutions S26
Figure S23. Specific conductivity of 2-PS-b-PAA-C12 and 3-PS-b-PAA-C12 solutions S27
Figure S24. Absorption spectra of Sudan I in 2-PS-b-PAA-C12 and 3-PS-b-PAA-C12 solutions S28
Figure S25. Absorption of Sudan I at λ = 485 nm in 2-PS-b-PAA-C12 and 3-PS-b-PAA-C12 solutions S29

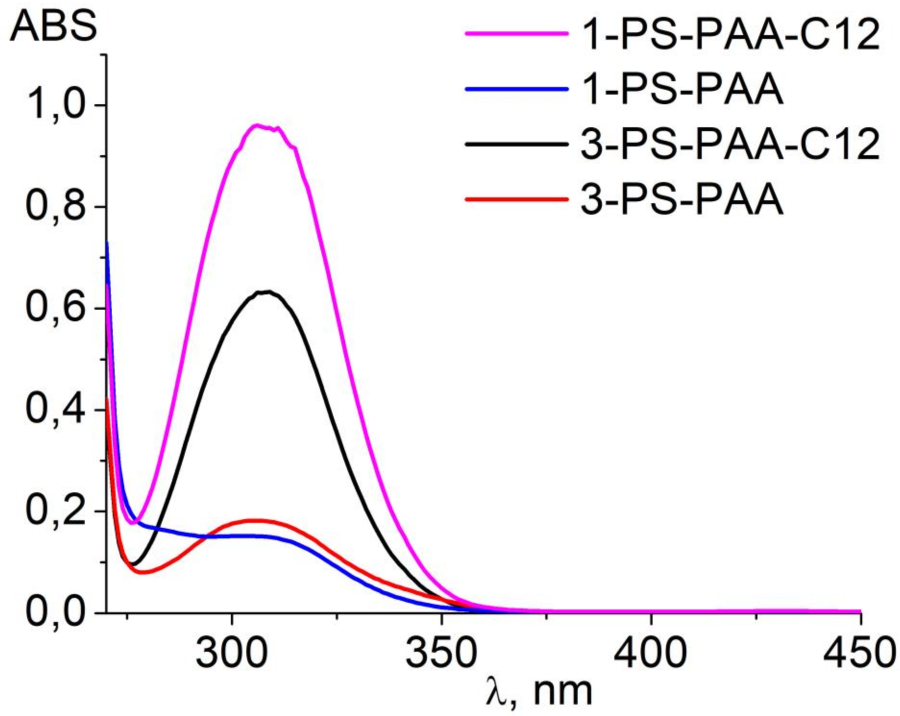

Figure S1. UV Absorbance spectra of 1-PS-b-PAA and 3-PS-b-PAA in DMF before and after hydrolysis of the trithiocarbonate group. The spectra are normalized to equal polymer concentrations.

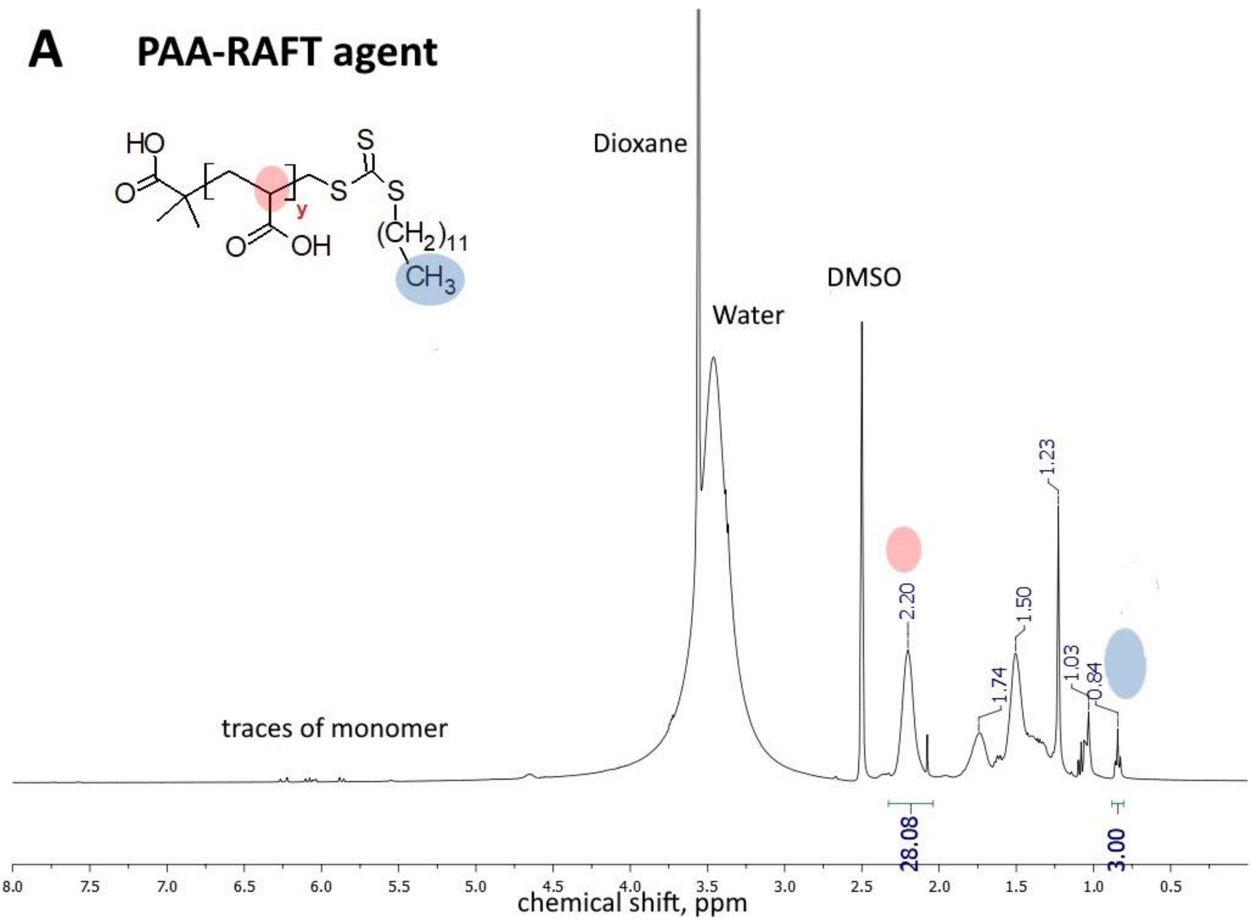

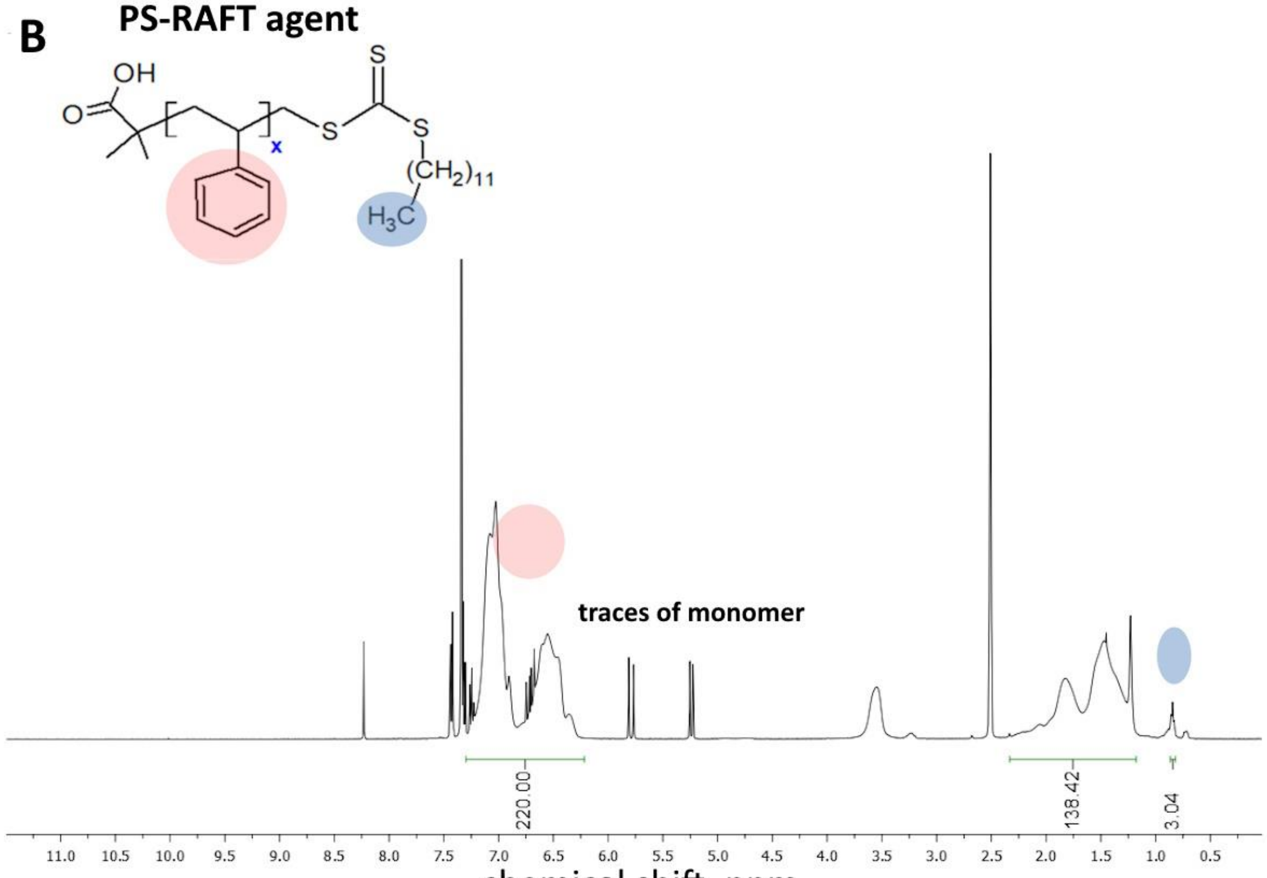

Figure S2. A- ^{1}H NMR spectra of macro-RAFT agent based on acrylic acid in DMSO-d6, B- ^{1}H NMR spectra of macro-RAFT agent based on styrene in DMSO-d6/$CDCl_3$ mixture

Table S1. Empty nanoreactor characteristics determined by DLS. Size is hydrodynamic diameter (particle number distribution), PDI is polydispersity index, and ξ or zeta potential is electrokinetic potential. The medium was water, pH = 7.4, 25 °C.

Type of copolymers	C (%, *w*/*w*)	Medium	T, °C	Size (nm)	PDI	ξ (mV)
3-PS-b-PAA	0.1	Tris HCl	25	59 ± 9	0.34 ± 0.02	-29.0 ± 1
3-PS-b-PAA	0.1	Tris HCl	37	68 ± 15	0.25 ± 0.02	-
3-PS-b-PAA	0.1	Tris HCl	45	190 ± 40	0.026 ± 0.02	-
3-PS-b-PAA	0.1	Tris HCl	55	190 ± 46	0.1 ± 0.03	-
1-PAA-b-PS	0.1	Tris HCl	25	28±5	0.44 ± 0.03	-24.0 ± 2
1-PAA-b-PS	0.1	Tris HCl	37	36 ± 10	0.37 ± 0.01	-
1-PAA-b-PS	0.1	Tris HCl	45	40 ± 2	0.42 ± 0.01	-
1-PAA-b-PS	0.1	Tris HCl	55	44 ± 1	0.42 ± 0.03	-

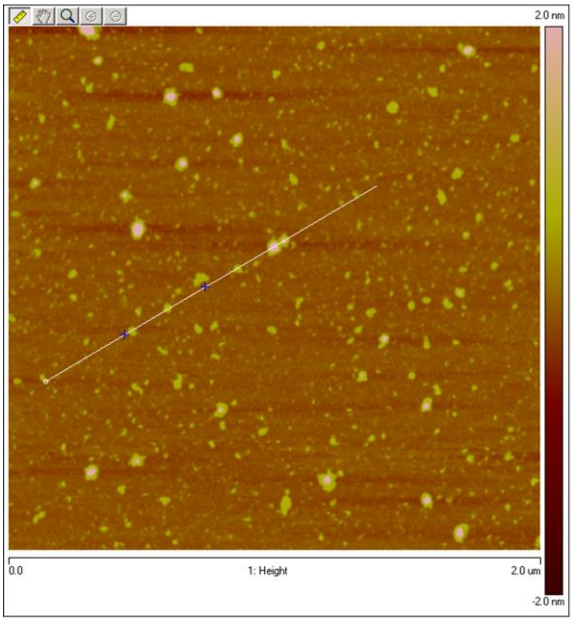

(a)

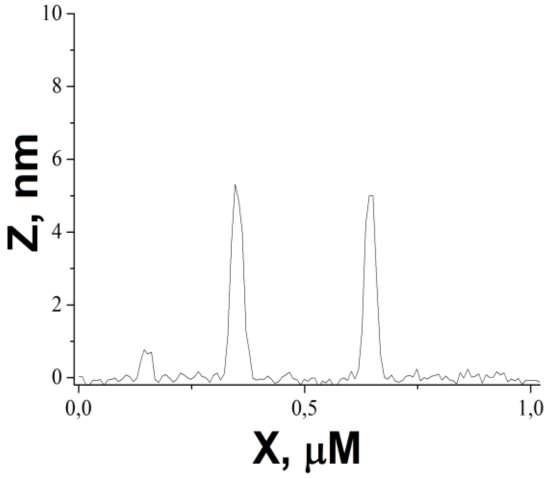

(b)

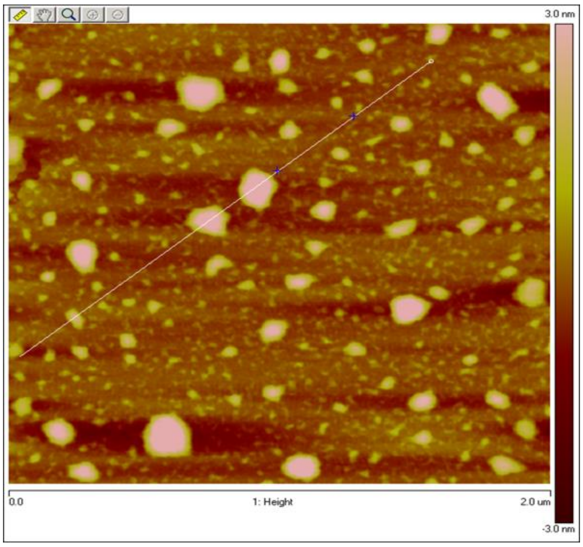

(c)

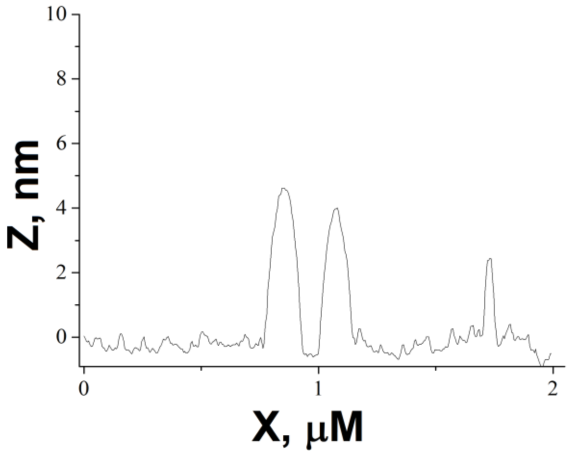

d)

Figure S3. AFM imaging of 1-PS-b-PAA (a, b) and 3-PS-b-PAA (c, d), $C_{Polymers}$ = 0.1%, water, 25 °C.

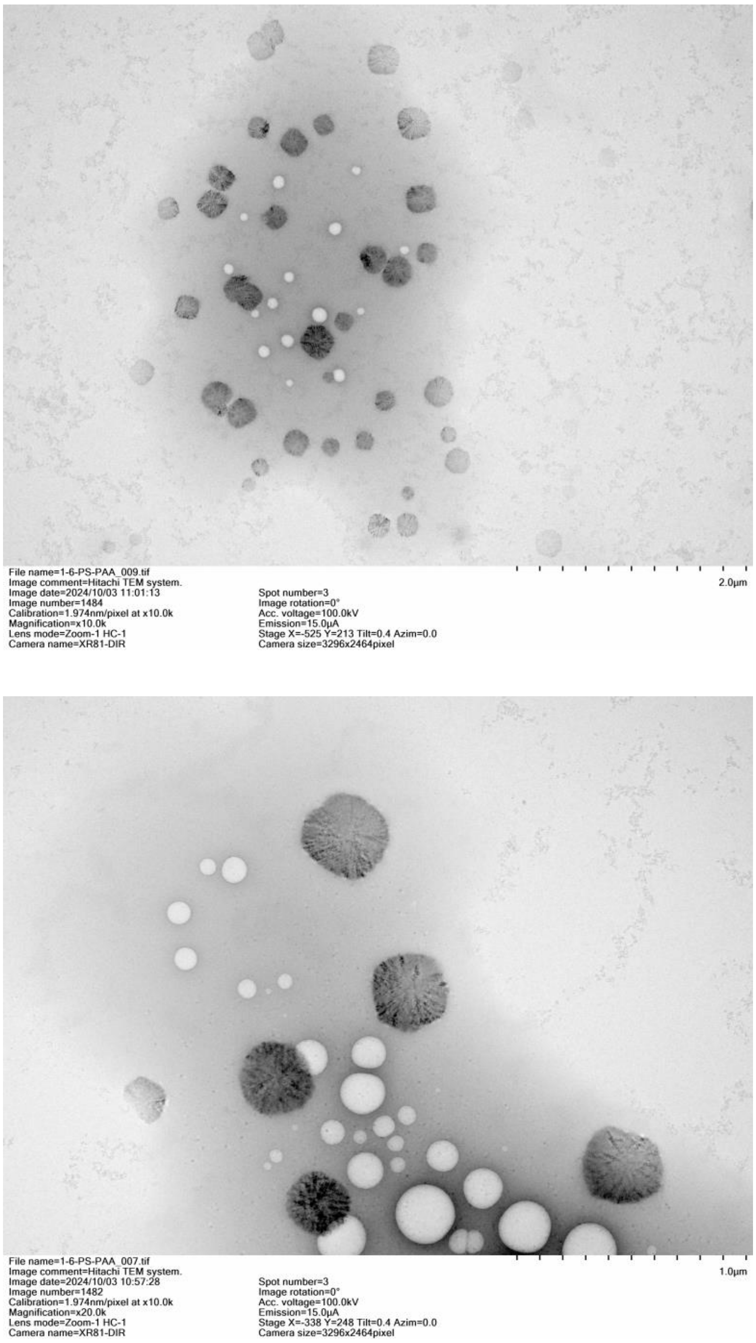

Figure S4. TEM imaging of BChE-loaded 1-PS-b-PAA, $C_{Polymers}$ = 0.01μg/mL, 10 mM TrisHCl-buffer, pH 7.4, 25 °C.

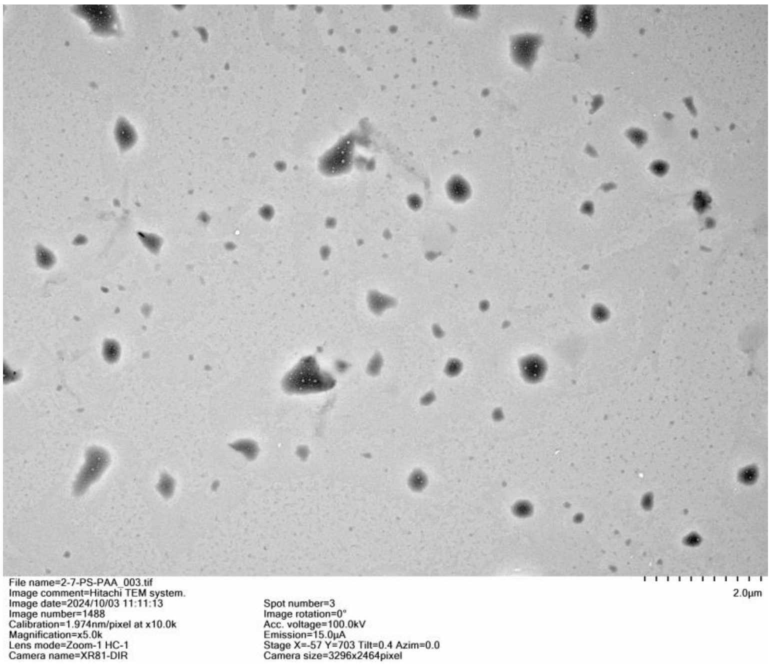

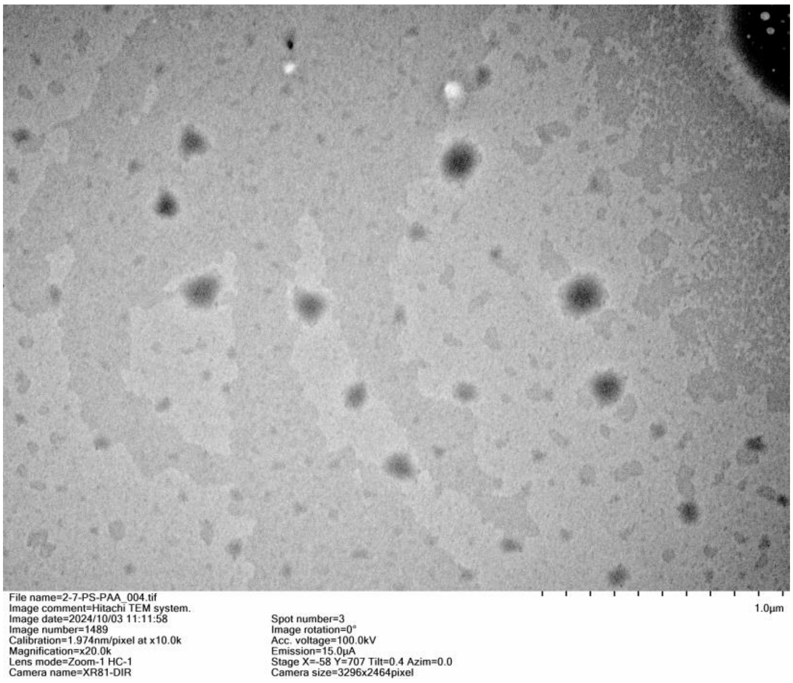

Figure S5. TEM imaging of BChE-loaded 2-PS-b-PAA, $C_{Polymers}$ = 0.01μg/mL, 10 mM TrisHCl-buffer, pH 7.4, 25 °C.

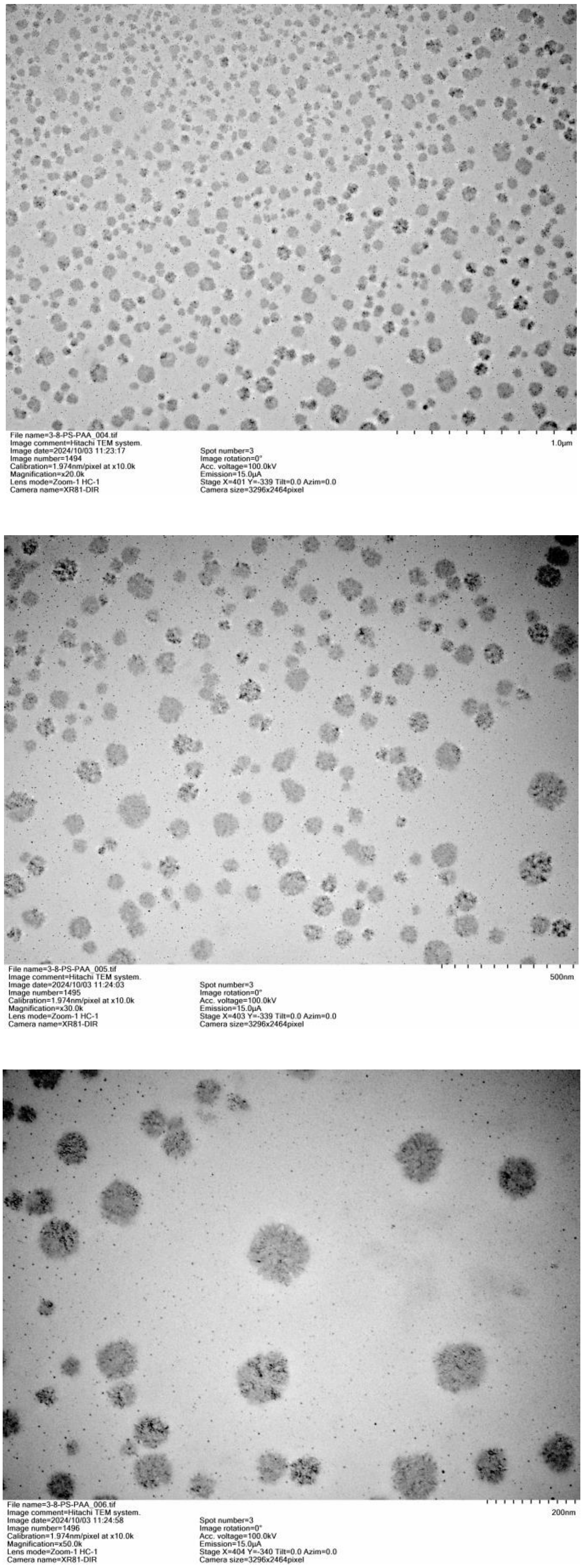

Figure S6. TEM imaging of BChE-loaded 3-PS-b-PAA, $C_{Polymers}$ = 0.01μg/mL, 10 mM TrisHCl-buffer, pH 7.4, 25 °C.

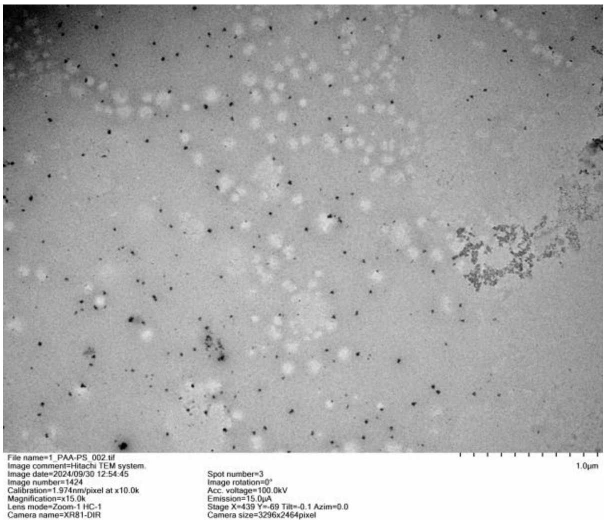

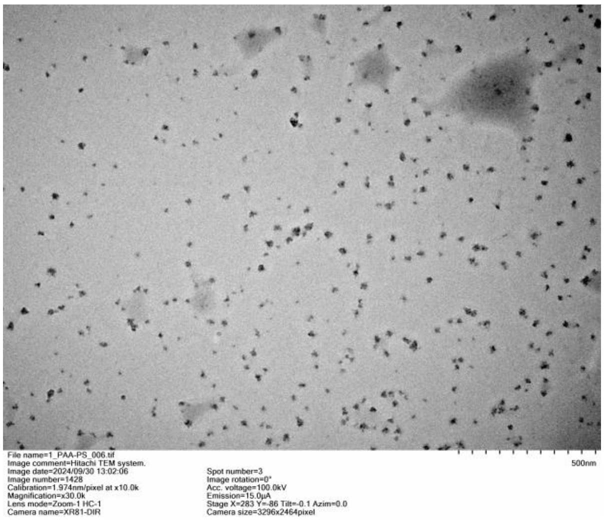

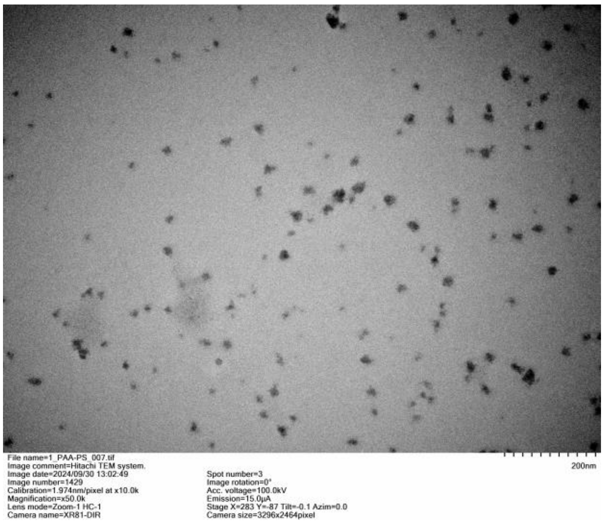

Figure S7. TEM imaging of BChE-loaded 1-PAA-b-PS, $C_{Polymers}$ = 0.01μg/mL, 10 mM TrisHCl-buffer, pH 7.4, 25 °C.

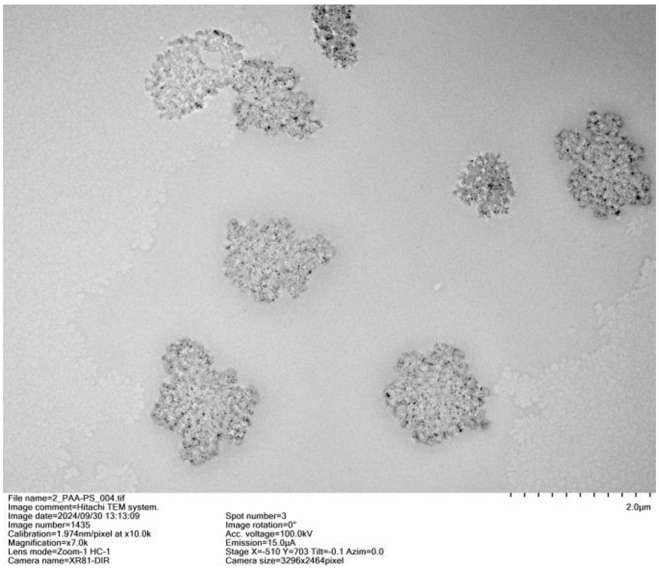

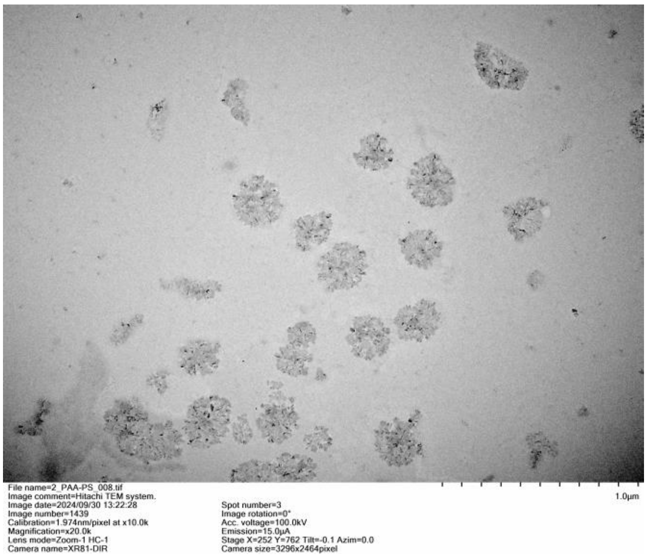

Figure S8. TEM imaging of BChE-loaded 2-PAA- b-PS, $C_{Polymers}$ = 0.01μg/mL, 10 mM TrisHCl-buffer, pH 7.4, 25 °C.

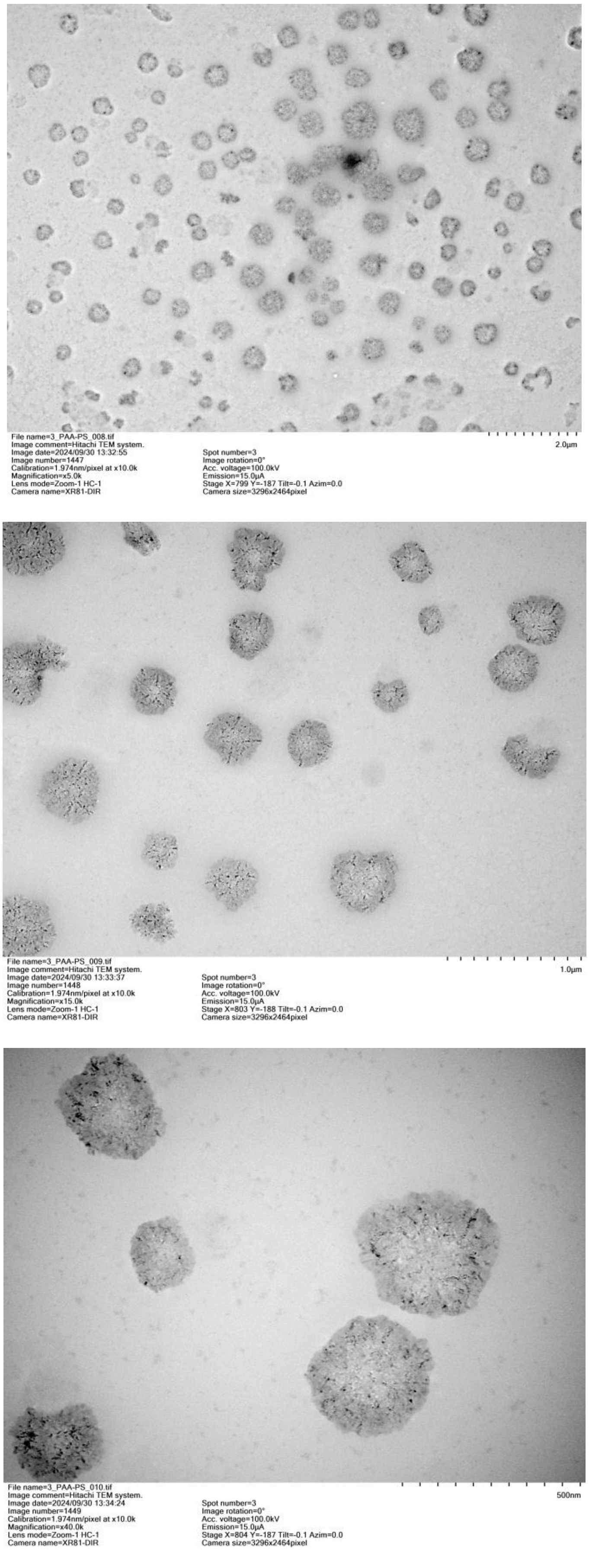

Figure S9. TEM imaging of BChE-loaded 3-PAA-b-PS, $C_{Polymers}$ = 0.01μg/mL, 10 mM TrisHCl-buffer, pH 7.4, 25 °C.

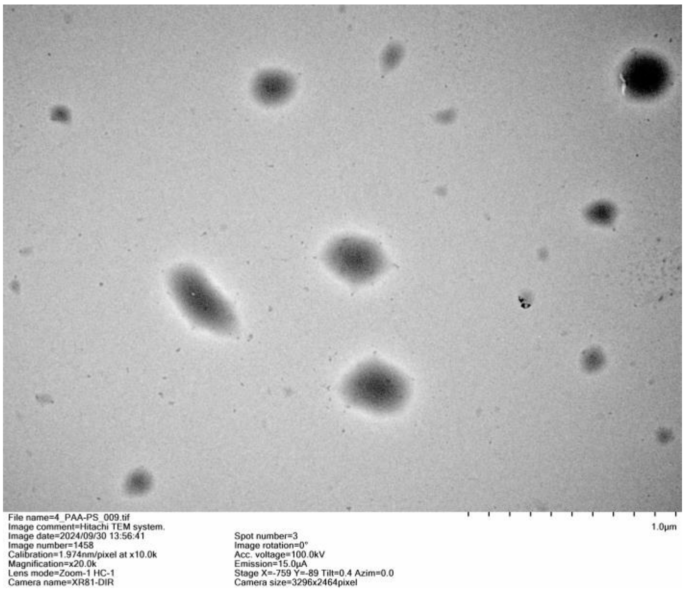

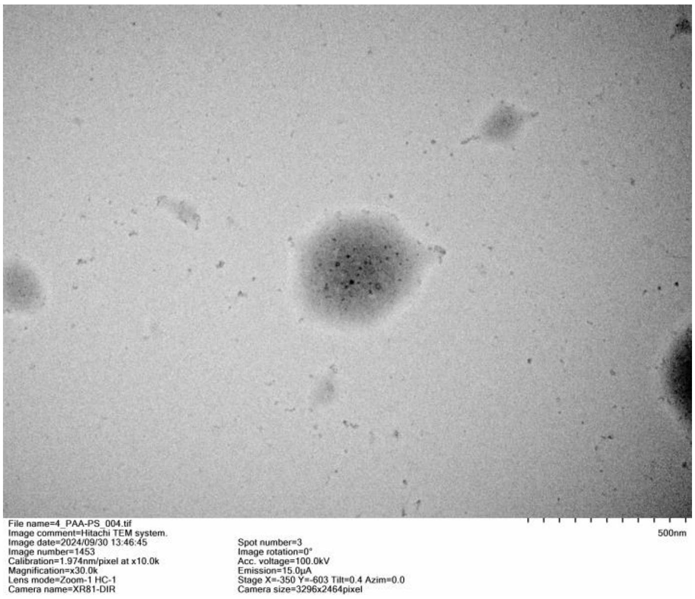

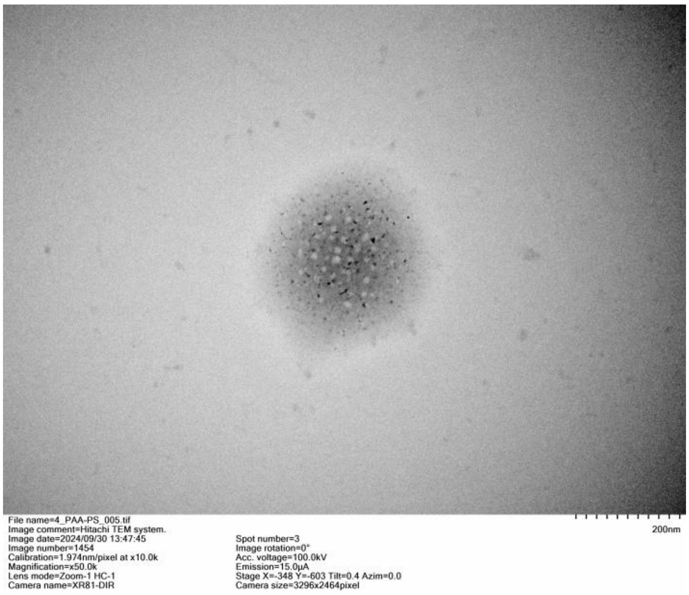

Figure S10. TEM imaging of BChE-loaded 4-PAA-b-PS, $C_{Polymers}$ = 0.01μg/mL, 10 mM TrisHCl-buffer, pH 7.4, 25 °C.

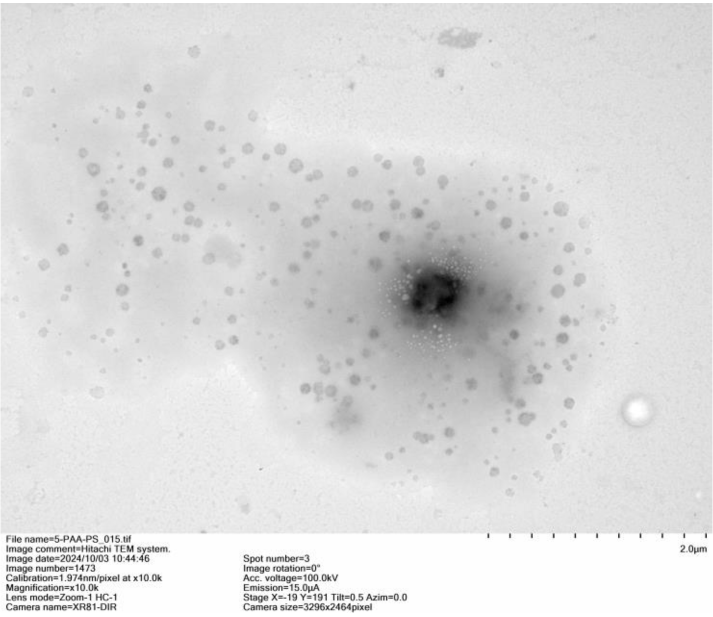

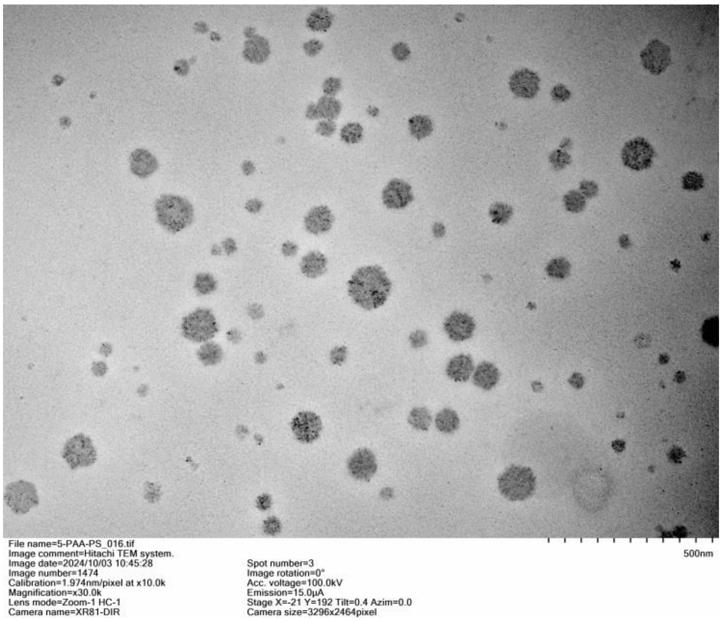

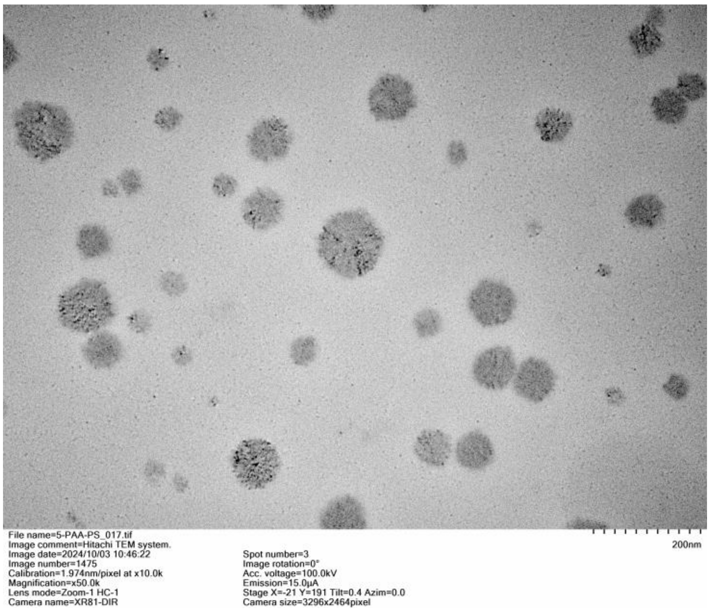

Figure S11. TEM imaging of BChE-loaded 5-PAA-b-PS, $C_{Polymers}$ = 0.01μg/mL, 10 mM TrisHCl-buffer, pH 7.4, 25 °C.

A

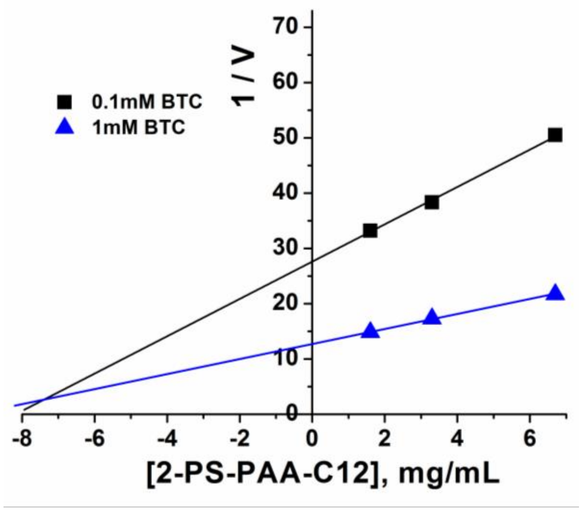

B

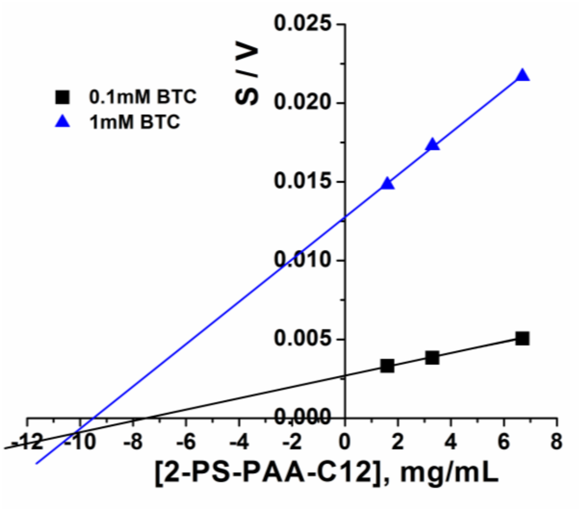

Figure S12. Dixon plot (A) and Cornish-Bowden plot (B) analyses of inhibition of BChE (0.0625 nM) by water-organic solution of 2-PS-b-PAA-C12 polymer, phosphate buffer 0.1 M, pH 7.0, 7.5% of MeOH, C_{BTC}=0.1, 1 mM, 25 °C.

A B

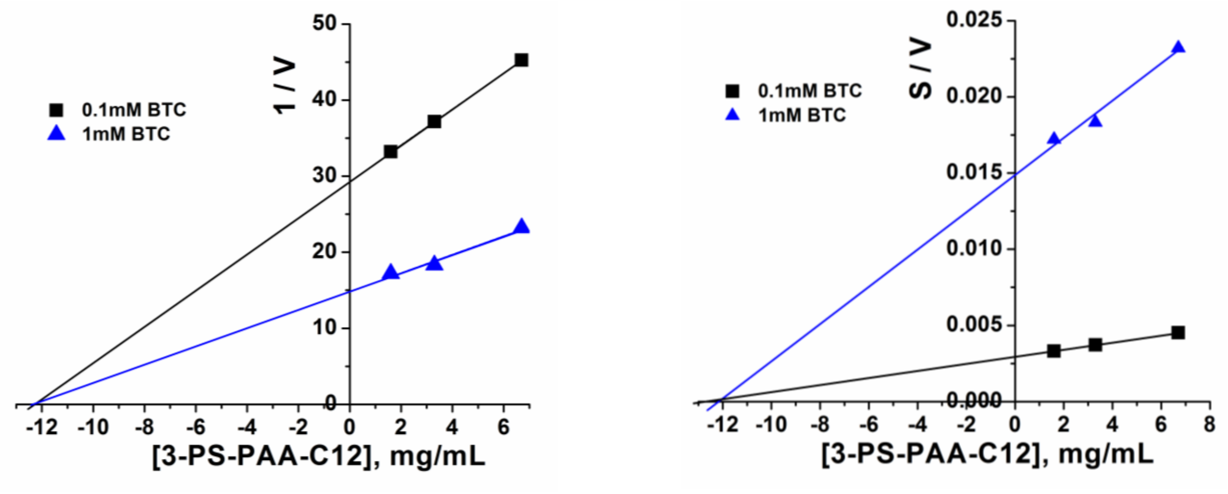

Figure S13. Dixon plot (A) and Cornish-Bowden plot (B) analyses of inhibition of BChE (0.0625 nM) by water-organic solution of 3-PS-b-PAA-C12 polymer, phosphate buffer 0.1 M, pH 7.0, 7.5% of MeOH, C_{BTC}=0.1, 1 mM, 25 °C.

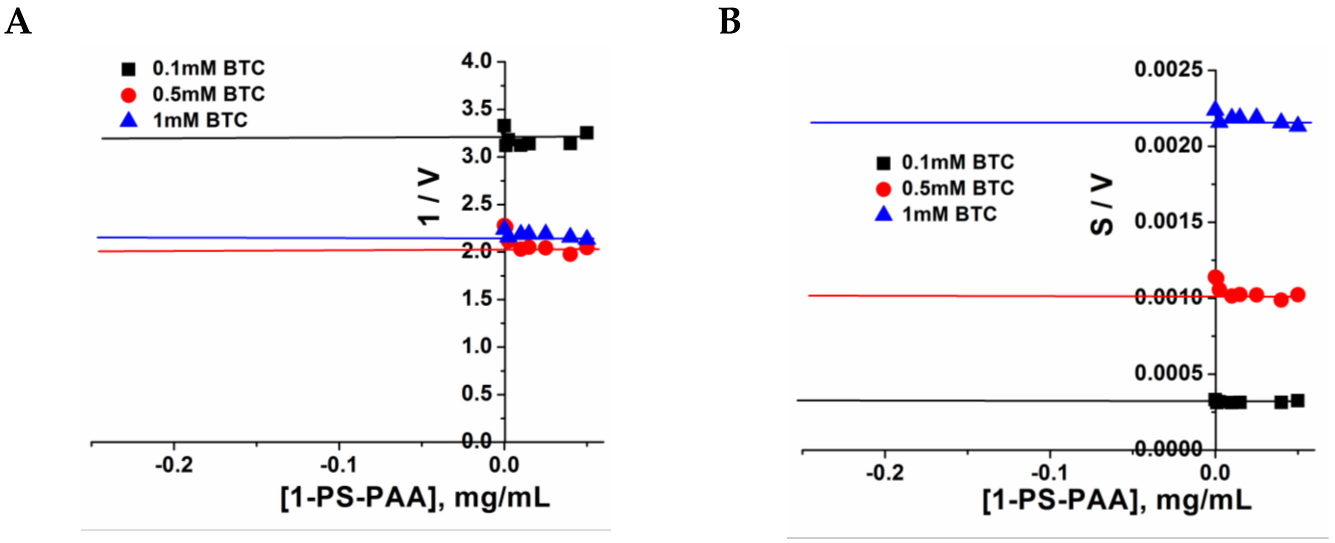

Figure S14. Dixon plot (A) and Cornish-Bowden plot (B) analyses of inhibition of BChE (0.625 nM) by 1-PS-b-PAA polymeric nanoparticles, phosphate buffer 0.1 M, pH 7.0, C_{BTC}=0.1, 0.5, 1 mM, 25 °C.

A

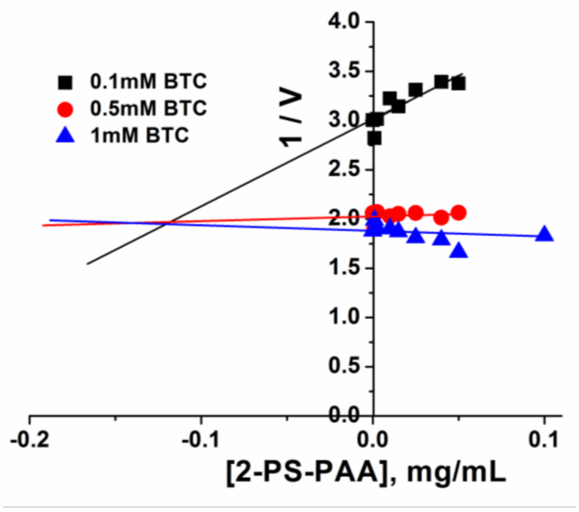

B

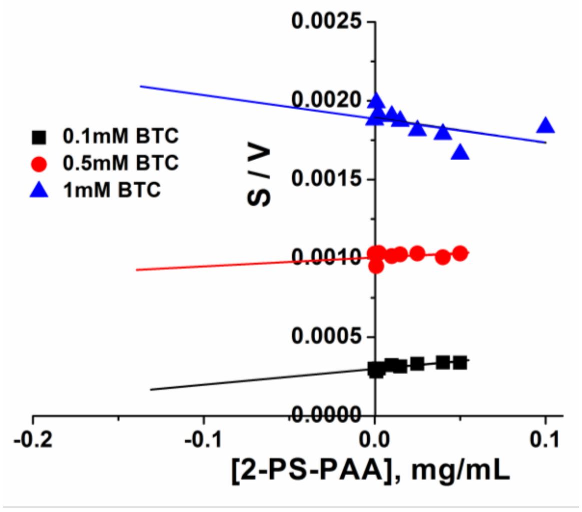

Figure S15. Dixon plot (A) and Cornish-Bowden plot (B) analyses of inhibition of BChE (0.625 nM) by 2-PS-b-PAA polymeric nanoparticles, phosphate buffer 0.1 M, pH 7.0, C_{BTC}=0.1, 0.5, 1 mM, 25 °C.

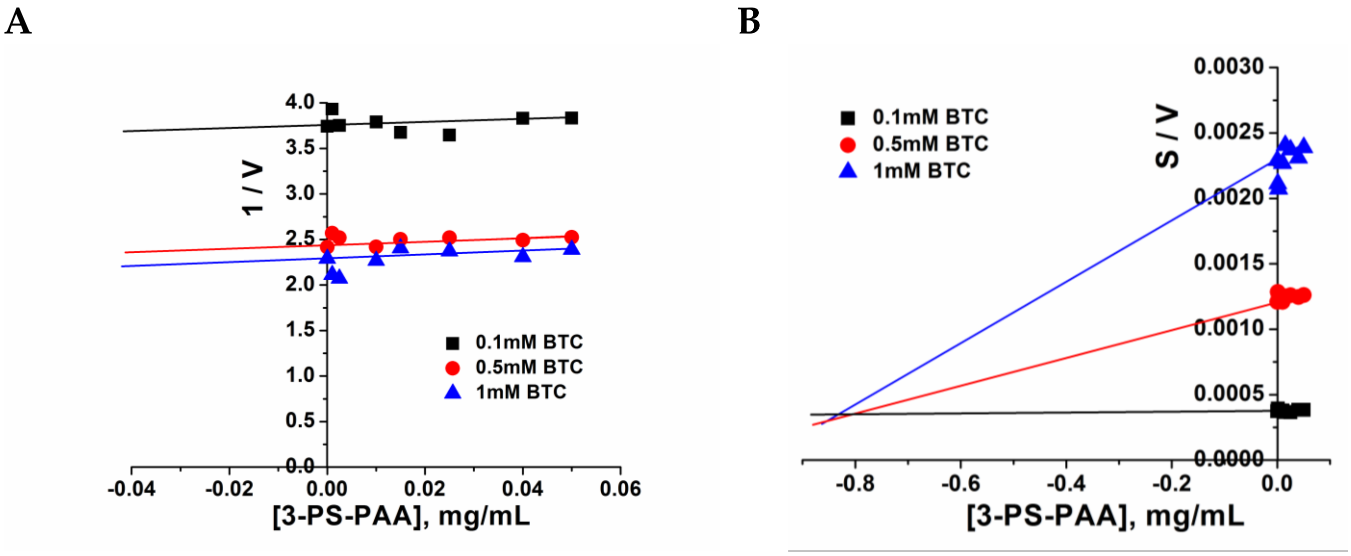

Figure S16. Dixon plot (A) and Cornish-Bowden plot (B) analyses of inhibition of BChE (0.625 nM) by 3-PS-b-PAA polymeric nanoparticles, phosphate buffer 0.1 M, pH 7.0, C_{BTC}=0.1, 0.5, 1 mM, 25 °C.

A B

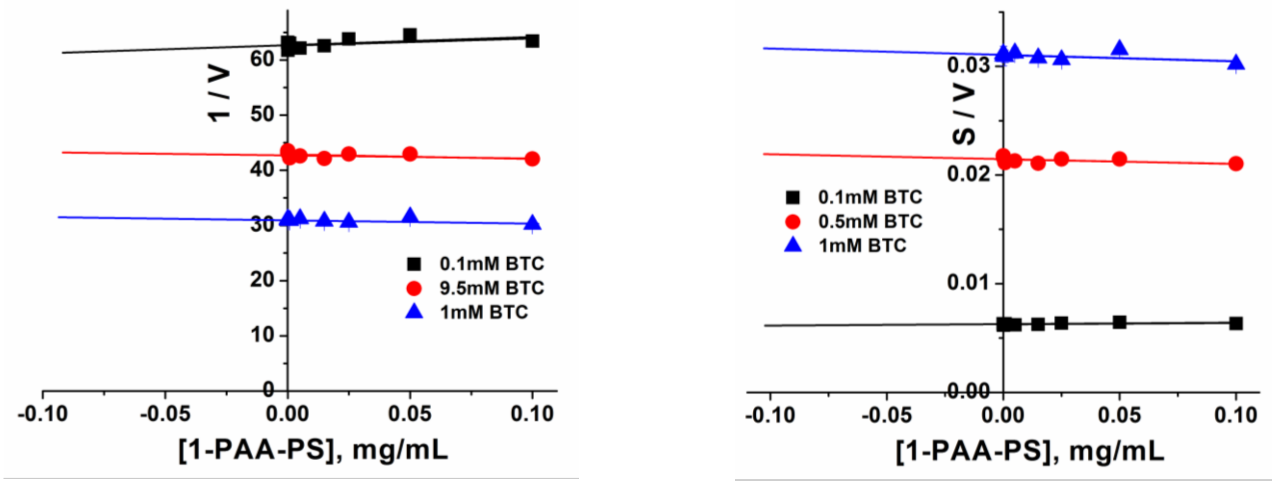

Figure S17. Dixon plot (A) and Cornish-Bowden plot (B) analyses of inhibition of BChE (0.05 nM) by 1-PAA-b-PS polymeric nanoparticles, phosphate buffer 0.1 M, pH 7.0, C_{BTC}=0.1, 0.5, 1 mM, 25 °C.

A

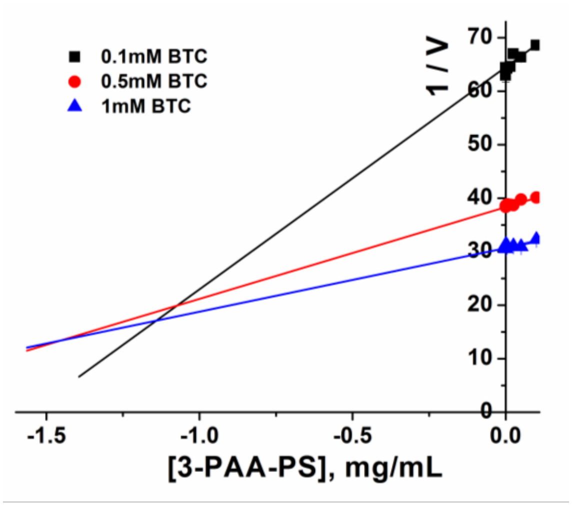

B

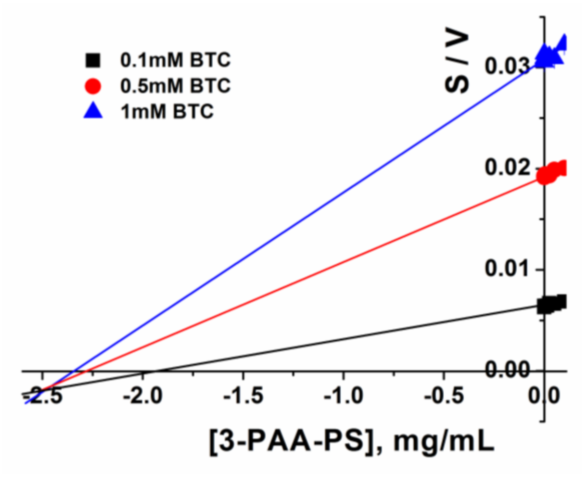

Figure S18. Dixon plot (A) and Cornish-Bowden plot (B) analyses of inhibition of BChE (0.05 nM) by 3-PAA-b-PS polymeric nanoparticles, phosphate buffer 0.1 M, pH 7.0, C_{BTC}=0.1, 0.5, 1 mM, 25 °C.

A B

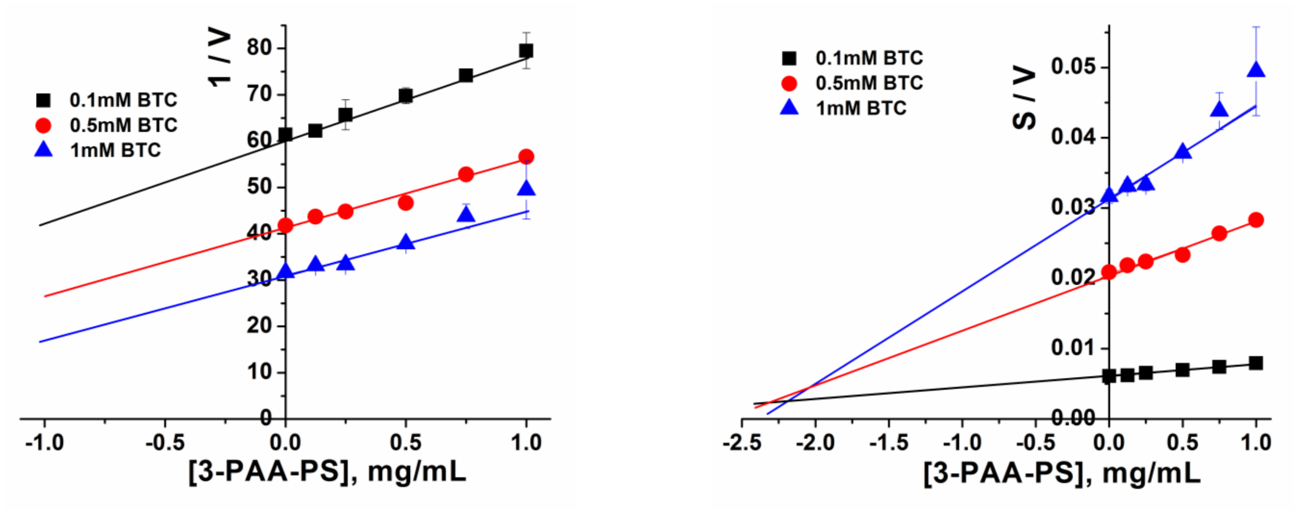

Figure S19. Dixon plot (A) and Cornish-Bowden plot (B) analyses of inhibition of BChE (0.05 nM) by water-organic solution of 3-PAA-b-PS polymer, 2% of DMSO, phosphate buffer 0.1 M, pH 7.0, C_{BTC}=0.1, 0.5, 1 mM, 25 °C.

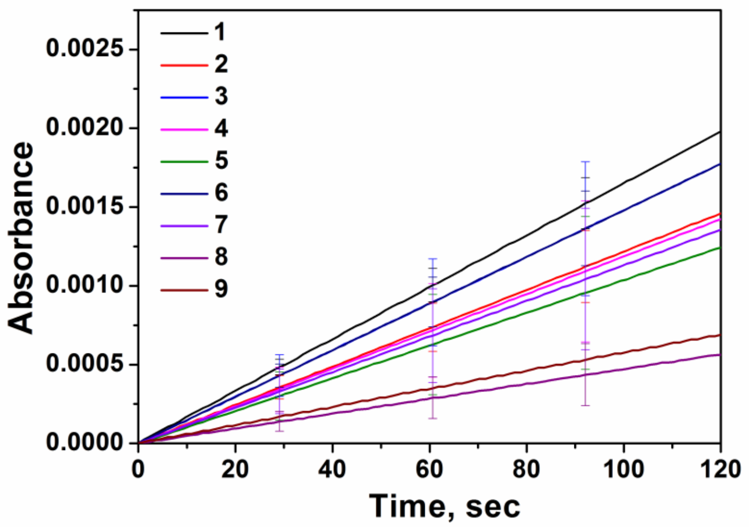

Figure S20. Progressive curves of spontaneous BTC hydrolysis in the presence of 3-PS-b-PAA after dilution. (1) Spontaneous hydrolysis of substrate BTC (2) after dilution 5 times (3), 10 times (4), 25 times (5), 50 times (6), 125 times (7) 250 times (8) and 500 times (9), Phosphate buffer 0.1 M, pH 7.0, C_{BTC}=1 mM, 25 °C

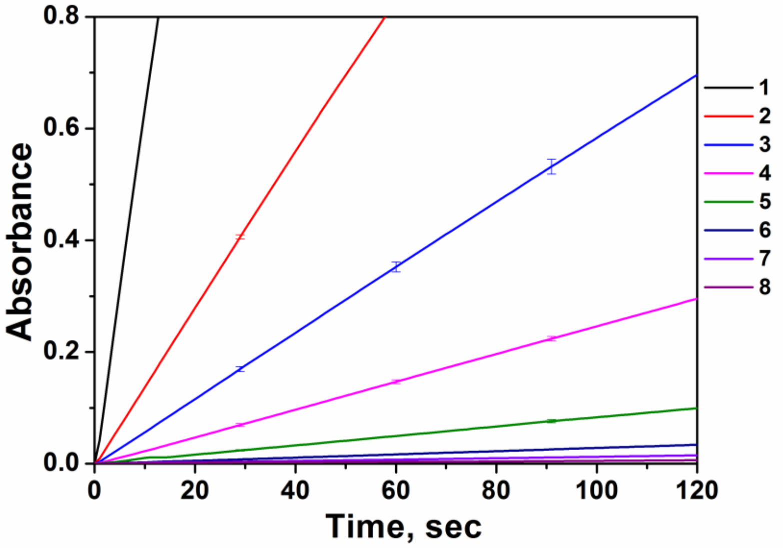

Figure S21. Progressive curves of free BChE-catalyzed hydrolysis of BTC (1), after dilution 5 times (2), 10 times (3), 25 times (4), 50 times (5), 125 times (6), 250 times (7), 500 times (8). Phosphate buffer 0.1 M, pH 7.0, C_{BTC}=1 mM 25 °C.

Table S2. Empty nanoreactor characteristics determined by DLS. The size is hydrodynamic diameter (particle number distribution), PDI is polydispersity index, and ξ or zeta potential is electrokinetic potential. The medium is water, 25 °C.

Type of copolymers	C (%, *w/w*)	Size (nm)	PDI	ξ (mV)
2-PS-b-PAA-C12	0.05	79±13	0.47 ± 0.03	-46 ± 1
	0.1	79±15	0.48± 0.04	-47 ± 1
	0.2	78±8	0.5 ± 0.03	-45 ± 1
	0.4	79±10	0.33 ± 0.04	-36 ± 1
	0.5	59±11	0.4± 0.02	-33 ± 1
3-PS-b-PAA-C12	0.05	68±14	0.42 ± 0.05	-45 ± 1
	0.1	68±14	0.42 ± 0.03	-38 ± 1
	0.2	59±11	0.43 ± 0.03	-37 ± 1
	0.4	79±12	0.38 ± 0.01	-37 ± 1
	0.5	91±23	0.56 ± 0.04	-29 ± 1

*extrusion

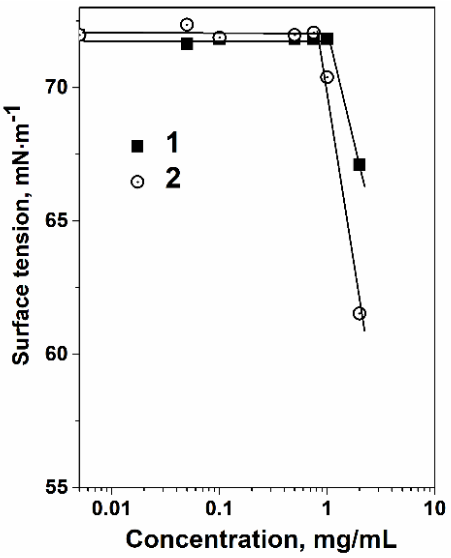

Figure S22. Surface tension of 2-PS-b-PAA-C12 (1) and 3-PS-b-PAA-C12 (2) block copolymers on their concentration, 25°C

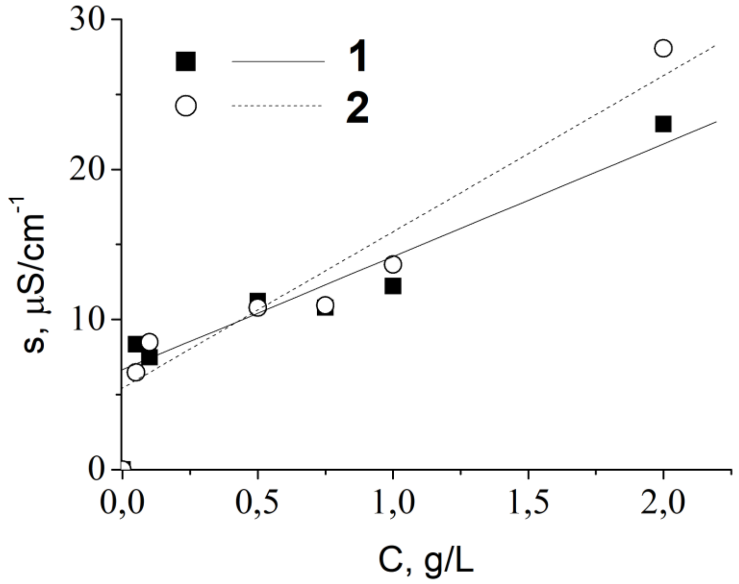

Figure S23. Specific conductivity of 2-PS-b-PAA-C12 (1) and 3-PS-b-PAA-C12 (2) block copolymer solutions on their concentration, 25°C

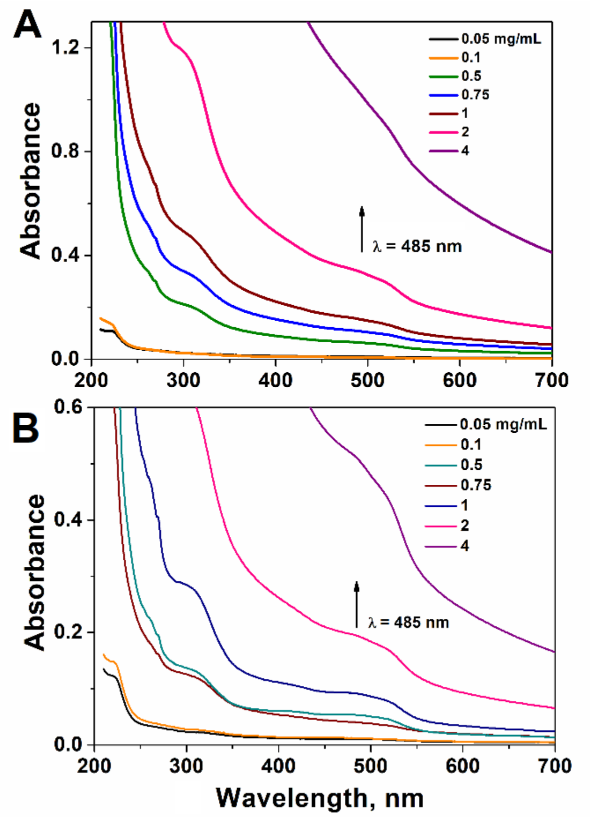

Figure S24. Absorption spectra of Sudan I in 2-PS-b-PAA-C12 (A) and 3-PS-b-PAA-C12 (B) block copolymer solutions on their concentration, L=0.2 cm, 25 ºC.

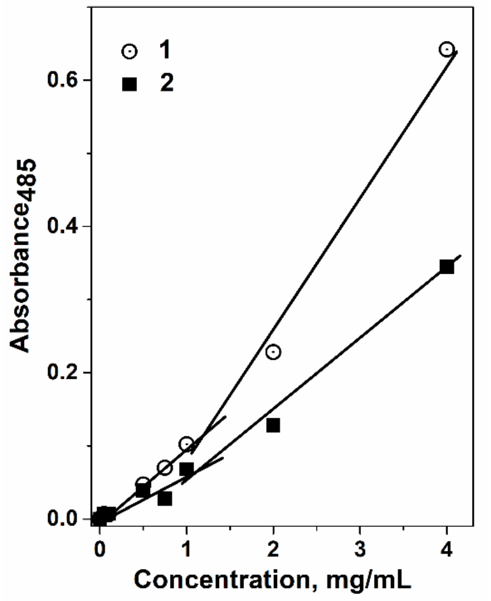

Figure S25. Absorption of Sudan I at λ = 485 nm in 2-PS-b-PAA-C12 (1) and 3-PS-b-PAA-C12 (2) block copolymer solutions on their concentration, L=0.2 cm, 25°C
